# A Novel Sol–Gel Synthesis Strategy of Co-Based Sillenite Composites for Enhanced Electrocatalysis of Water Splitting

**DOI:** 10.1021/acsomega.5c09012

**Published:** 2026-02-12

**Authors:** Mayara Acioli, Leandro Bufaiçal, P. R. A. de Oliveira, Yngrid Synara de Sena Silva, Liying Liu, Ana Luisa Silva, Nakédia M. F. Carvalho

**Affiliations:** † 28130 Universidade do Estado do Rio de Janeiro, Instituto de Química, Rio de Janeiro 20550-013, Brazil; ‡ Universidade Federal de Goiás, Instituto de Física, Goiânia 74690-900, Brazil; § 28125 Universidade Federal do Rio de Janeiro, Instituto de Física, Rio de Janeiro 21941-909, Brazil; ∥ 74350 Centro Brasileiro de Pesquisas Físicas, Rio de Janeiro 22290-180, Brazil

## Abstract

This work reports a new synthetic strategy and investigates the effect of thermal treatment on Bi and Co oxide composites (BCO), comprising Co-based sillenite (Bi_24–*x*_Co_*x*_)­Co_2_O_40_, Co_3_O_4_, and Bi_2_O_3_, as well as their application as electrocatalysts in the oxygen evolution reaction (OER) and hydrogen evolution reaction (HER). The materials were synthesized by a modified Pechini sol–gel method using citric acid as a complexing agent and ethylene glycol as a polymerization agent and were calcined in the range between 500 and 1000 °C. Rietveld refinement of the XRD data confirmed the predominance of the sillenite and Co_3_O_4_ crystalline phases, both with cubic structures, with minor quantities of α-Bi_2_O_3_ (monoclinic) at 500 and 600 °C. A higher sillenite content (>60 wt %) was observed at 700 and 800 °C, while at 900 °C the sillenite phase decreased, disappearing completely at 1000 °C, where α-Bi_2_O_3_ became dominant. The BCO sample calcined at 700 °C exhibited the lowest overpotentials for both the OER and HER in 1 mol L^–1^ KOH (pH 14), 475 and 307 mV at j ± 10 mA cm^–2^, respectively. The corresponding Tafel slopes for the OER and HER of 62 and 23 mV dec^–1^, respectively, also exhibited the lowest values. The superior catalytic performance of BCO-T700 can be related to the synergy between Co^2+^ and Bi^3+^ species shown by X-ray photoelectron spectroscopy. These findings reinforce the potential of sillenite-based composites as efficient, low-cost, bifunctional electrocatalysts for water splitting and hydrogen production. Notably, no prior electrochemical studies of cobalt sillenites synthesized via the adapted Pechini method were found, emphasizing the relevance of this work.

## Graphical abstract

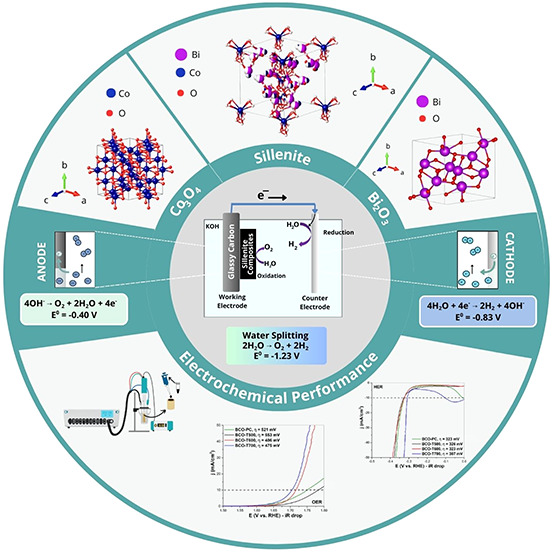

## 1 Introduction

The prolonged use of fossil fuels has caused significant environmental impacts, particularly due to the unbalanced emission of greenhouse gases.^1,2^ Much effort has been focused on the adoption of more sustainable and cleaner sources of energy for industrial and consumer applications, exploring renewable raw materials (biomass, bioalcohols, and water) as well as primary renewable energy sources, such as solar, wind, tidal, or hydroelectric power.^3^ Green hydrogen produced by water electrolysis is a promising energy carrier that meets the technological requirements to mitigate the aforementioned issues.^4^

In the water electrolysis process, an energy input is required (Δ*G*° = +237.18 kJ mol^–1^), and the reaction, known as water splitting, results in the production of oxygen gas at the anode through the oxygen evolution reaction (OER). Simultaneously, hydrogen gas is produced at the cathode through the hydrogen evolution reaction (HER).^5,6^ The OER is the limiting step of water electrolysis due to its sluggish kinetics; however, catalysts are required for both OER and HER. Noble metal oxides, such as IrO_2_ and RuO_2_, are the most widely used commercial electrocatalysts for OER, while Pt is the benchmark catalyst for HER.^7^ However, despite their high efficiency, large-scale application is limited by the high cost and low abundance of these elements.^8,9^

A promising alternative involves the first-row transition-metal-based materials, such as Co, Fe, and Ni, particularly their metal oxides, as substitutes for the noble-metal catalysts.^10^ These materials exhibit suitable conductivity and stability, efficient charge transfer, and high to moderate electrochemical performance, in addition to being more accessible and abundant in nature.^8^ For instance, cobalt oxides are widely used in electrochemical applications, being efficient as electrodes for low-cost fuel cells and water electrolysis.^11^ However, some Co-based advanced materials, such as perovskites, are difficult to synthesize under mild pressure and temperature, the solid-state being the most adopted method despite being highly energy-consuming and dependent on expensive reactors that operate at high temperature and pressure. When attempts are made to synthesize Co-perovskites under mild conditions, other crystalline structures, such as sillenites, are often formed.^12^

Sillenites are crystalline bismuth-based oxides isostructural to γ-Bi_2_O_3_ that can be doped with numerous chemical elements, whose ideal stoichiometric structures can be represented as Bi_12_MO_20±δ_ or Bi_24_M_2_O_40±δ_, where M (transition metal) can be a divalent, trivalent, tetravalent, or pentavalent ion.^12–15^ Furthermore, efforts are also directed toward exploring the sillenites of new structures and stoichiometries. It is important to highlight that structural changes in the sillenite caused by doping can increase the electrical conductivity, thus improving charge mobility.^14^ Although numerous sillenite syntheses have been recently reported, Co-based sillenite has remained relatively less explored. Nonetheless, the few available studies employ different synthetic approaches such as solid-state routes – Bi_12_(Bi_0.55_Co_0.45_)­O_19.6_,^16^ hydrothermal methods – Bi_10_Co_16_O_38_–Bi_25_FeO_40_,^17^ and sol–gel methods – (Bi_13_Co_12_)­CoO_40_ and (Bi_13_Co_5.5_Fe_6.5_)­CoO_40_.^18^

Recently, multication oxides of the sillenite type have been applied in several energy-related topics, such as electrocatalysis,^7,10^ photocatalysis,^19,20^ and photoelectrocatalysis.^21,22^ The search for catalysts that exhibit enhanced activity, low overpotentials, and long-term stability has motivated interest in sillenite-type oxides. For instance, Vijay et al.^10^ reported that the Fe-based sillenite Bi_25_FeO_40_ exhibited an overpotential of 640 mV vs RHE at a current density of 1 mA cm^–2^ for the OER in 0.1 mol L^–1^ KOH. Similarly, Arora et al.^7^ reported that another Fe-based sillenite Bi_24_Fe_2_O_39_ achieved a lower overpotential of 420 mV at 10 mA cm^–2^ in 1 mol L^–1^ KOH, thereby underscoring the catalytic potential of this class of materials. Nevertheless, no reports were found regarding the application of Co-doped sillenites in either the OER or HER. In contrast, other Bi–Co oxide systems, such as the perovskites BiCoO_3_ ^23^ and (Bi_0.5_Co_0.5_)_2_O_3_^24^ have demonstrated excellent OER performance, with 303 mV and 367 mV overpotential at 10 mA cm^–2^, respectively.

This work presents the evaluation of the bifunctional efficiency of Co-based sillenite for both OER and HER electrocatalysis. A new synthetic strategy was employed, based on the adapted Pechini method, using citric acid and ethylene glycol as precursors, followed by an investigation of the influence of the calcination temperature on the samples. The crystalline phases, electronic structures, morphologies, and electrochemical properties of the synthesized composites were investigated. The efficiency and stability of the materials in water-splitting electrocatalysis were explored in an alkaline medium, and the postmortem characterization of the catalysts was provided. This is the first report on the use of this type of oxide composite applied as an electrocatalyst for water splitting.

## 2 Experimental Section

### 2.1 Materials

For the synthesis procedures, the following reagents were used without further purification: Bi­(NO_3_)_3_·5H_2_O (≥98.0%, Sigma-Aldrich), Co­(NO_3_)_2_·4H_2_O (98.0%, Sigma-Aldrich), citric acid (≥99.5%, Sigma-Aldrich), and ethylene glycol (99.5%, Sigma-Aldrich). All aqueous solutions were prepared with ultrapure water. Additional analytical-grade chemicals and reagents were employed as received: KOH (90.0%, Sigma-Aldrich), RuO_2_ (99.9%, Sigma-Aldrich), Nafion (technical grade, Toronto Research Chemicals Inc.), 1-propanol (≥99.8%, Tedia), isopropyl alcohol (≥99.8%, Sigma-Aldrich), and methanol (99.5%, Dinâmica). A stock solution of 5% w/v Nafion (250 mg of Nafion in 0.75 mL of ultrapure water, 2 mL of isopropyl alcohol, 2 mL of 1-propanol, and 0.25 mL of methanol) was prepared for further use in the electrode film preparation. Fluorine-doped tin oxide (FTO) glass substrates (7 Ω cm^–2^, Sigma-Aldrich) with dimensions of 1 × 4 cm were cleaned by sequential sonication in distilled water with 2% v/v Extran (Merck), followed by ethanol and acetone (100%, Tedia) for 10 min each and subsequently dried at room temperature.

### 2.2 Synthesis of Co-Based Sillenite Composites

Co-based sillenite composites (BCO) were synthesized via a sol–gel route adapted from the Pechini method.^25,26^ A solution of the metal nitrate precursors, citric acid (CA), and ethylene glycol (EG) was prepared in a molar ratio of Bi:Co:CA:EG of 1:1:5:4. In brief, 10.003 g of Bi­(NO_3_)_3_·5H_2_O (20.62 mmol), 6.006 g of Co­(NO_3_)_2_·4H_2_O (20.62 mmol), and 20.001 g of CA (104 mmol) were dissolved in 100 mL of ultrapure water. Subsequently, EG (4.50 mL, 80 mmol) was added to the solution. The resulting reddish milky mixture was stirred under reflux at 70 °C for 5 h. The obtained gel was dried in a muffle furnace at 150 °C for 10 h by using a heating rate of 20 °C min^–1^. The solid foam formed was ground and precalcined at 300 °C for 2 h (heating rate of 2 °C min^–1^). To evaluate the effect of thermal treatment, the powder was further calcined for 4 h (heating rate of 2 °C min^–1^) at different temperatures: 500, 600, 700, 800, 900, and 1000 °C. After cooling, the samples were ground in an agate mortar and stored for further characterization and electrochemical analysis. The samples were named BCO-T, where T is the treatment temperature (Table S1).

### 2.3 Characterization of Co-Based Sillenite Composites

Thermogravimetric analysis (TA Instruments Model SDT Q600) was performed in the temperature range of 30–1000 °C (heating rate of 10 °C min^–1^) under a synthetic air flow (150 mL min^–1^) to investigate the thermal stability of the samples. X-ray diffraction (XRD, Bruker D8 ADVANCE diffractometer) patterns of the composite powders were measured over a 2θ range of 10°–80°, with a step size of 0.02° and a scan rate of 0.04° min^–1^ (Cu Kα radiation, λ = 1.5406 Å). The phase identification and their mass percentages were determined by Rietveld refinement via the general structure analysis system (GSAS) software and the EXPGUI graphical interface.^27^ The morphology and microstructure of the samples were examined by using field-emission scanning electron microscopy (SEM, JEOL JSM-7100F) and field-emission transmission electron microscopy (TEM, JEOL JEM-2100F). The chemical composition was determined by energy-dispersive X-ray spectroscopy (EDS). For TEM analysis, the samples were prepared by drop-casting an aqueous suspension of the material onto carbon-coated copper grids, followed by drying under air. Fourier transform infrared (FTIR) vibrational spectra were acquired in the wavenumber range of 2000–400 cm^–1^ using KBr pellets on a PerkinElmer Frontier spectrometer. Raman spectroscopy (Horiba Scientific XploRA Plus confocal Raman microscope) was performed with a 785 nm excitation source from an Ar+ laser, at 3.5% power in a backscattering configuration, equipped with a CCD detector. Elemental quantification of the digested samples was carried out using an inductively coupled plasma optical emission spectrometer (ICP-OES, iCAP 6000 series, Thermo Scientific) equipped with a Mira Mist nebulizer (Agilent Technologies) and a cyclonic spray chamber. Argon (≥99.95%) was used as the nebulizer gas. The operational parameters included a radiofrequency power of 1300 W and a nebulizer gas flow rate of 0.75 L min^–1^. The emission lines monitored were 223.0 and 238.8 nm for Bi and Co, respectively. X-ray photoelectron spectroscopy (XPS) measurements were performed in an ultrahigh-vacuum chamber with a base pressure of approximately 8 × 10^–10^ mbar, equipped with a PHOIBOS 150 hemispherical analyzer (SPECS) providing an overall energy resolution of about 0.7 eV. A nonmonochromatic Al Kα X-ray source (hν = 1486.6 eV) operated at 100 W was used, yielding a sample current below 1 nA. All spectra were recorded at room temperature using a 60° takeoff angle to enhance surface sensitivity, over an analyzed area of approximately 1–2 mm^2^. Survey spectra and high-resolution core-level spectra were acquired with pass energies of 50 and 30 eV, respectively. The spectrometer energy scale was calibrated against the Au 4f_7/2_ photoemission line at 84.0 eV. Spectral deconvolution and quantification were carried out using CasaXPS software, employing a Shirley background and fitting the peaks with a mixed Gaussian–Lorentzian GL­(x) line shape, with x = 50 providing the lowest residuals across all analyses.^28^ Quantification was based on the relative sensitivity factors (RSFs) following the Scofield table and the inelastic mean free path (IMFP) appropriate for each element and energy region considered.^29,30^

### 2.4 Electrochemical Measurements

Electrochemical analyses were carried out using an Autolab PGSTAT302N potentiostat/galvanostat (Metrohm), controlled via NOVA 2.1.7 software. A conventional cell consisting of three electrodes was employed, comprising a saturated calomel electrode (SCE, 3 mol L^–1^ KCl) as the reference electrode, a platinum bar as the counter electrode, and a glassy carbon (GC) or FTO electrode modified with the sillenite composite films as the working electrode (WE). The GC electrode was coupled to a rotating disc electrode (RDE) system (AUT.RDE, Metrohm) operating at 1600 rpm. All measurements were conducted in an alkaline medium, 1 mol L^–1^ of KOH at pH 14.

Catalyst inks were prepared by dispersing 16 mg of composite powder in 2 mL of ultrapure water, followed by 30 min in the ultrasonic bath to ensure a homogeneous suspension.^31^ For the GC electrode, 20 μL of the ink was deposited on the surface by drop-casting to achieve a catalyst loading of 0.8 mg cm^–2^.^32^ After drying, 5 μL of the stock solution of 5% w/v Nafion diluted in isopropyl alcohol in a 1:30 volume ratio was added dropwise over the catalyst layer. For the FTO electrodes, 102 μL of the ink was deposited in a 1 cm^2^ area, followed by drying and application of 20 μL of the same diluted Nafion solution. All electrodes were dried in air prior to the electrochemical measurements. It should be noted that for the materials calcined at 800–1000 °C, catalyst ink preparation was not possible, as they failed to form a homogeneous suspension due to sintering during high-temperature calcination. All electrochemical measurements were performed with the GC RDE electrode, except for the overall water splitting, electrochemical impedance spectroscopy (EIS), and chronopotentiometry analyses, which were performed with the FTO. For chronopotentiometry, an additional annealing step of the films at 300 °C for 1 h (heating rate of 20 °C min^–1^) was carried out to improve adhesion to the FTO substrate.

For the characterization of the redox peaks, experiments of cyclic voltammetry (CV) were carried out at 100 V s^–1^ varying the potential from −0.43 to 1.57 V (vs RHE).

For the double-layer capacitance (C_dl_) determination, CV measurements were carried out in the non-Faradaic region, varying the scan rate from 10 to 1000 mV s^–1^. The capacitive current (*i_C_*) was derived from anodic and cathodic responses, and *C*_*dl*_ was calculated from the slope of the linear plot of *i_C_* vs the scan rate (ν), according to eq 1.^33^

1 $$i_{C}=C_{dl}\cdot v$$

The electrochemically active surface area (ECSA) of the electrodes was estimated as defined in eq 2. The ECSA (cm^2^) was obtained by the division of *C_dl_* by specific capacitance (*C_s_*, mF cm^–2^), considering the value of 0.040 mF cm^–2^ in an alkaline medium.^34,35^

2 $$\mathrm{ECSA}=\frac{C_{dl}}{C_{s}}$$

The surface roughness factor (RF) was obtained by normalizing ECSA by the geometric area of the electrode, where *S*_geo_ is 1 cm^2^ for the FTO, as expressed by eq 3:^34,36^

3 $$\mathrm{RF}=\frac{\mathrm{ECSA}}{S_{\mathrm{geo}}}$$

Electrochemical impedance spectroscopy (EIS) measurements were conducted using a frequency range of 10^5^ to 0.1 Hz and voltage amplitude of 0.01 VRMS AC amplitude at 1.57 V vs RHE. The Nyquist and Bode plots were fitted using an equivalent circuit model in the Nova 2.1.7 software. The effective capacitance (*C*_eff_) of the electrode was estimated based on the parameters of the fitted constant phase element (CPE), as described in eq 4, where it is required to know the solution resistance (*R_S_*), the magnitude of the CPE admittance (*y*_0_) and the index “*N”* which measures how closely the CPE, with values between 0 and 1, approaches an ideal capacitor.^37^

4 $$C_{\mathrm{eff}}=\frac{{\left(y_{0}\cdot R_{S}\right)}^{1/N}}{R_{S}}$$

### 2.5 Water-Splitting Electrocatalysis

Electrocatalytic activity for OER and HER was assessed by linear sweep voltammetry (LSV), from 1.47 to 2.0 V vs RHE under a scan rate of 5 mV s^–1^ for OER, and from 0.57 to −0.63 V vs RHE under a scan rate of 5 mV s^–1^ for HER. An ohmic drop (*iR*) correction (85%) was applied to all measurements, using GC RDE as WE. Each analysis was performed in duplicate, and the voltammograms presented correspond to the averaged data, which were expressed as current density by normalizing the measured current (i, A) to the geometric surface area of the electrode (*S*_geo_, cm^2^), according to eq 5:^24^

5 $$j=\frac{i}{S_{\mathrm{geo}}}\times 1000$$

The applied potentials were converted from SCE to the reversible hydrogen electrode (RHE) scale, as shown in eq 6:^24^

6 $$E_{\mathrm{RHE}}=E_{\mathrm{applied}}+E_{\mathrm{SCE}}^{0}+\left(0.059\times \mathrm{pH}\right)$$

The overpotentials (η) for the OER and HER were calculated according to eqs 7 and 8, respectively.^24,34,38^

7 $$\eta_{\mathrm{OER}}=E_{\mathrm{RHE}}-1.23V$$

8 $$\eta_{\mathrm{HER}}=\left|E_{\mathrm{RHE}}-0\right|V$$

Tafel slopes were determined by linear fitting of the logarithmic current density vs overpotential obtained from LSV using eq 9:^34,39^

9 η=blogj+a

The specific activity (SA, mA cm^–2^) was calculated by dividing the current *i* (mA) at *η* = 450 mV for OER and 300 mV for HER by the ECSA, as observed in eq 10:^34^

10 $$SA_{\eta }=\frac{\left(i_{\eta }\right)}{\mathrm{ECSA}}$$

The stability of the materials was evaluated by chronopotentiometry at a current density of 10 mA cm^–2^ for OER and at −5 mA cm^–2^ for HER, using FTO as the working electrode (WE). For OER, the Faradaic efficiency (ηF) was measured using a single-compartment cell with a coupled polarographic dissolved oxygen probe (Hanna Instruments, HI6421P-02) and with a two-compartment H-type cell of 2 × 25 mL with a Nafion proton-permeable membrane, where the headspace oxygen gas was measured by volumetry. The η_F_ accounted for both the dissolved oxygen and the headspace. For HER, the evolved gas was measured by using a two-compartment H-type cell by volumetry. The Faradaic efficiency was determined using eq 11 and compared with the theoretical value derived from the Faraday equation:

11 $$\eta_{F}=\frac{n_{02\mathrm{measured}}}{n_{02\mathrm{calculated}}}\times 100$$

The overall water splitting test was carried out using a two-electrode cell under alkaline conditions (1 mol L^–1^ KOH, pH 14), with FTO as the substrate and at the same catalyst loading (0.8 mg cm^–2^). The catalysts were used simultaneously as the anode and cathode, varying the potential from 0 to 2.25 V (OER) and from −0.8 to −2.25 V (HER).

## 3 Results and Discussion

### 3.1 Characterization of the Co-Based Sillenite Composites

Thermogravimetric analysis (TGA) (Figure 1) was employed to investigate the thermal decomposition of the precursors synthesized via the Pechini method using Bi­(NO_3_)_3_.5H_2_O and Co­(NO_3_)_2_.4H_2_O as metal sources, with citric acid and ethylene glycol as chelating and polymerization agents, respectively, after the drying step at 150 °C. The thermogram exhibited multiple mass-loss stages, which were attributed to the removal of adsorbed and crystallization water, degradation of the polymeric matrix, decomposition of nitrate species, and the eventual formation of metal oxides. The first stage, occurring up to approximately 150 °C, corresponds to humidity removal, with a mass loss of ∼5 wt %, consistent with dehydration behavior reported for similar systems.^40,41^ The second stage, centered around 302 °C, marks the onset of degradation of the polyester matrix formed by citric acid and ethylene glycol, along with partial decomposition of the nitrate species, leading to oxynitrate intermediates such as BiONO_3_.^42,43^ This step resulted in a significant mass loss of 43 wt %. A subsequent, more intense mass-loss event, centered at 349 °C and corresponding to approximately 26 wt % mass loss, likely reflects the complete decomposition of the remaining nitrates and advanced pyrolysis of the organic framework, accompanied by the release of NO_2_ and CO_2_. The fourth stage, observed near 368 °C, is probably associated with the transformation of oxynitrates into stable metal oxides, with a mass loss of 1.8 wt %. Finally, an additional mass-loss event was detected around 915 °C, which may be attributed to partial volatilization of bismuth,^43^ as suggested by the XRD, ICP-OES and SEM results discussed below, oxygen loss resulting in suboxide formation, or combustion of residual carbon. According to these results, a precalcination step at 300 °C was added to remove part of the organic materials before the calcination at 500–1000 °C, which helped in the handling and grinding of the solid and avoided excessive sintering compared to when the calcination was carried out directly.

**Figure 1:**
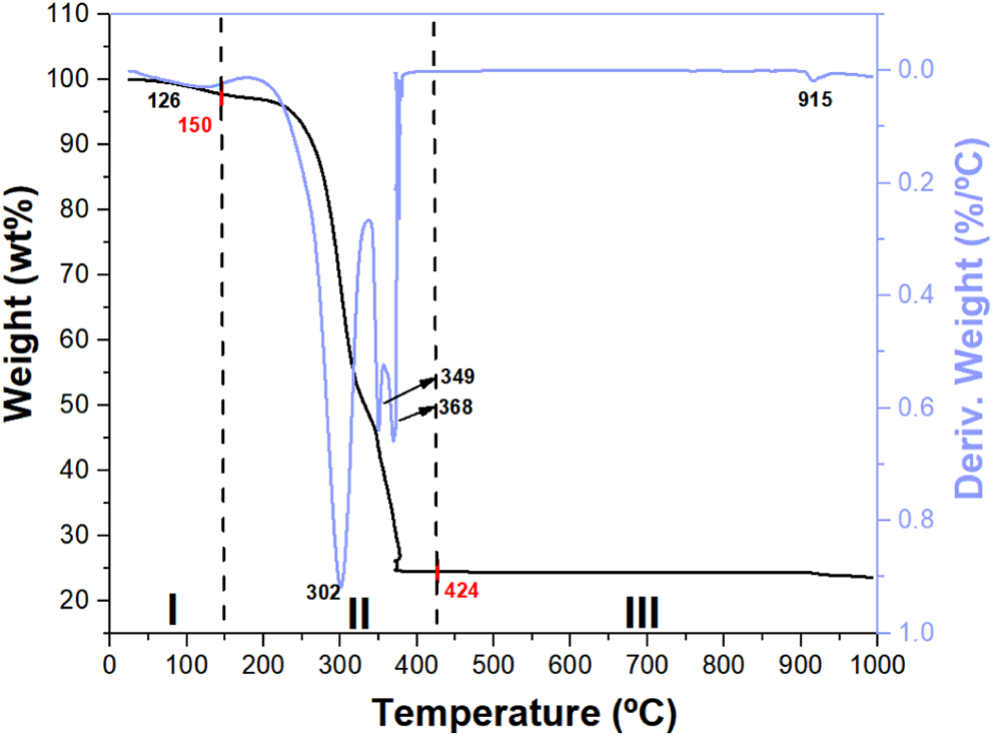
TGA/DTG analysis of the sillenite composite precursor gel, previously dried in a muffle furnace at 150 °C for 10 h (heating rate: 20 °C min^–1^), was conducted at 10 °C min^–1^ under a synthetic air flow (150 mL min^–1^).

The elemental composition of the samples was determined by ICP-OES (Table S2). For the samples calcined between 500 and 700 °C, the data showed a Bi:Co molar ratio of roughly 1:1, a value consistent with the nominal ratio employed in the synthesis, indicating that the sol–gel method was effective in incorporating the metallic precursors into the final composition without losses or enrichment of a specific element in the samples. However, above 700 °C, a gradual increase in Co content in relation to bismuth was observed, which is attributed to the volatilization of bismuth during the calcination step.^12^ For the estimation of the chemical formula of the composites, the oxidation states of the elements were assumed to be Bi^3+^, Co^3+^, and O^2–^.

Room temperature XRD was employed to verify the crystalline structures present in the BCO samples and to determine the composition of the phases. Figure 2 shows the resulting diffractograms. As can be seen, the precalcinated (BCO-PC) sample exhibits low-intensity and undefined peaks, some of which remain unindexed with respect to any of the considered crystal phases, namely sillenite, Co_3_O_4_ and Bi_2_O_3_. These features are indicative of incomplete crystallization of the material due to the residue of CA and EG during thermal treatment at 300 °C, as evidenced by the TGA results. For BCO-T500, the Bragg peaks are more intense and well-defined, signaling the improvement of the system’s crystallinity. From the Rietveld refinement (Figure S1 and Table 1), BCO-T500 is composed of approximately 17, 35, and 48 wt % of the (Bi_18_Co_16_)­Co_2_O_40_ (sillenite), Co_3_O_4_ and α-Bi_2_O_3_ phases, respectively. Increasing the calcination temperature to 600–800 °C leads to an enhancement of the sillenite phase at the cost of a significant decrease in the α-Bi_2_O_3_ phase, as depicted in Table 1. Further increase in the calcination temperature leads to enhancement of the sillenite phase, while the Co_3_O_4_ phase vanishes.

**Figure 2:**
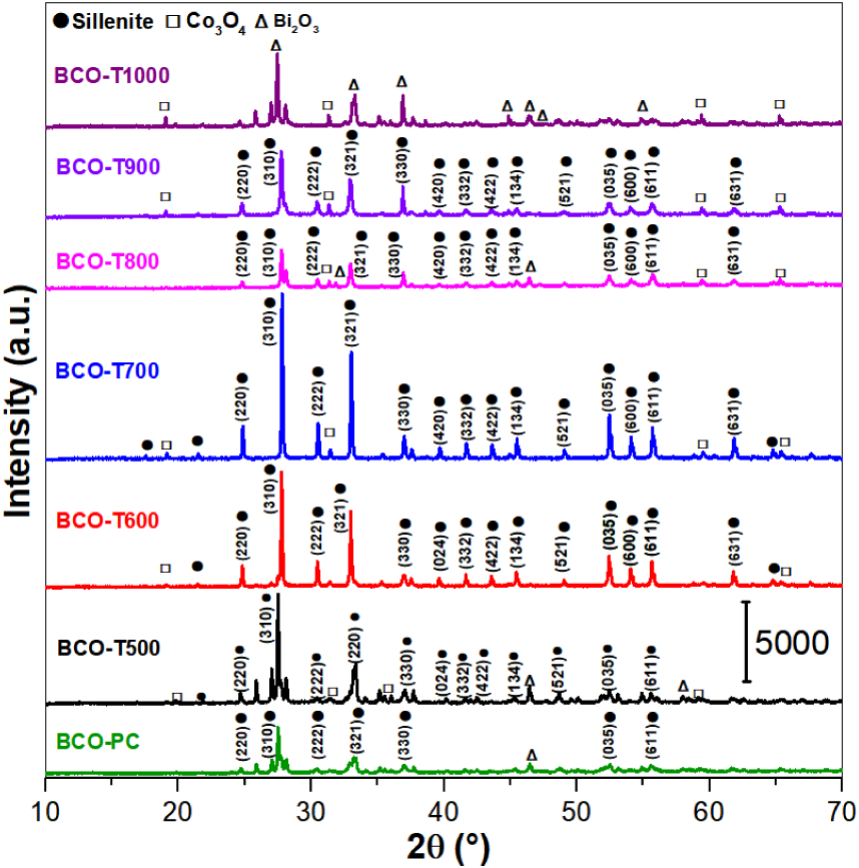
X-ray diffractograms of the BCO samples at different calcination temperatures.

**Table 1:** Structural Parameters Obtained from Rietveld Refinement and Phase Agreement Factors of the BCO Materials

**Samples**	**Phase**	**Proportion** **(wt %)**	**Symmetry**	**Space groups**	***a*** (A)	***b*** (A)	***c*** (A)	***χ*** ^**2**^	***R***_**wp**_ **(%)**	***R***_**p**_ **(%)**
BCO-T500	(Bi_18_Co_16_)Co_2_O_40_	17	Cubic	*I23*	10.2082(6)	10.2082(6)	10.2082(6)	1.2	4.4	3.5
	Co_3_O_4_	35	Cubic	*Fd-3m*	8.0829(6)	8.0829(6)	8.0829(6)			
	α-Bi_2_O_3_	48	Monoclinic	*P2*_*1*_ */c*	5.8516(2)	8.1666(2)	7.5118(3)			
BCO-T600	(Bi_18_Co_6_)Co_2_O_40_	54	Cubic	*I23*	10.1905(2)	10.1905(2)	10.1905(2)	1.5	5.3	4.1
	Co_3_O_4_	41	Cubic	*Fd-3m*	8.0839(5)	8.0839(5)	8.0839(5)			
	α-Bi_2_O_3_	5	Monoclinic	*P2*_*1*_ */c*	5.8517(11)	8.1652(11)	7.5124(12)			
BCO-T700	(Bi_18_Co_6_)Co_2_O_40_	63	Cubic	*I23*	10.1893(1)	10.1893(1)	10.1893(1)	1.6	4.6	3.6
	Co_3_O_4_	37	Cubic	*Fd-3m*	8.0847(2)	8.0847(2)	8.0847(2)			
BCO-T800	(Bi_12_Co_12_)Co_2_O_40_	69	Cubic	*I23*	10.1838(4)	10.1838(4)	10.1838(4)	1.3	5.6	5.7
	Co_3_O_4_	24	Cubic	*Fd-3m*	8.0831(3)	8.0831(3)	8.0831(3)			
	β-Bi_2_O_3_	7	Tetragonal	*P-4b2*	10.9144(9)	10.914(9)	5.6304(7)			
BCO-T900	(Bi_16_Co_8_)Co_2_O_40_	56	Cubic	*I23*	10.1824(6)	10.1824(6)	10.1824(6)	1.3	4.3	3.5
	Co_3_O_4_	40	Cubic	*Fd-3m*	8.0859(4)	8.0859(4)	8.0859(4)			
	β-Bi_2_O_3_	4	Tetragonal	*P-4b2*	10.9293(16)	10.9293(16)	5.6206(13)			
BCO-T1000	α-Bi_2_O_3_	63	Monoclinic	*P2*_*1*_ */c*	5.8515(3)	8.1630(4)	7.5086(5)	1.4	4.1	3.2
	Co_3_O_4_	37	Cubic	*Fd-3m*	8.0850(2)	8.0850(2)	8.0850(2)			

Interestingly, when a calcination temperature of 800 °C is adopted, approximately 7 wt % of a β-phase of Bi_2_O_3_ appears. Bismuth oxide is known to exhibit an extraordinarily rich phase polymorphism, denoted by α (monoclinic), β (tetragonal), γ (cubic, bcc), δ (cubic, fcc), ε (orthorhombic), and ω (triclinic) phases.^44,45^ When produced at temperatures below 700 °C, α-Bi_2_O_3_ is formed, while above 730 °C the δ-phase appears. Through the cooling of δ-Bi_2_O_3_ from high temperature to room temperature, a large thermal hysteresis effect is formed, with the possible occurrence of intermediate metastable phases that can be stabilized at room temperature, depending on the cooling rate and/or the presence of other elements.

For BCO-900, the percentages of both the sillenite and β-Bi_2_O_3_ phases decrease compared to those of BCO-800, while the portion of Co_3_O_4_ naturally increases. Finally, for BCO-1000, both the sillenite and β-Bi_2_O_3_ phases vanish, and the material is composed of 63% α-Bi_2_O_3_ and 37% Co_3_O_4_. The overall XRD results further evidence that this system is highly sensitive to the synthesis conditions.^46–48^

Detailed microstructural characterization is shown in Figure 3, and the BCO materials consist of agglomerated particles, likely comprising sillenite, Co_3_O_4_, and Bi_2_O_3_ grains. The samples display irregularly shaped particles at both micro- and nanoscale, with morphological features changing with calcination temperature. The BCO-PC, BCO-T500, BCO-T600, and BCO-T700 samples exhibited an irregular morphology surrounded by irregular nanopolyhedra particles up to ∼500 nm in size, likely originating from nanoparticle coalescence during thermal treatment. This morphology is consistent with that reported for (Bi_13_Co_11_)­Co_2_O_40_–Co_3_O_4_ materials synthesized via the sol–gel method.^12,15^ A similar structural arrangement was reported by Wu et al. for Bi_12_SiO_20_ prepared via a modified Pechini method and calcined at 630 °C.^19^ Underscoring the evolution of structural features across different length scales at 800 °C and above, extensive sintering was observed, leading to the formation of thick, irregular plates, extending to several tens of micrometers and exhibiting surface grooves, particularly in BCO-T900 and BCO-T1000. Although groove features are not typically associated with sillenite phases, similar structures have been reported for BiCoO_3_ synthesized via solid-state reactions.^49^ Grain growth in the range of 1–2 μm was also evident, likely resulting from prolonged high-temperature annealing. The presence of cavities is attributed to the removal of volatile species during calcination, while agglomeration becomes more pronounced at elevated temperatures. The strong agglomeration observed in these samples further indicates effective powder sintering, consistent with the findings of Mokrý et al.^50^ XRD analysis of BCO-T1000 indicated the predominance of α-Bi_2_O_3_, which typically exhibits plate-like morphology with heterogeneous particle sizes when obtained by sol–gel synthesis.^51^ Co_3_O_4_ prepared via a low-temperature hydrothermal route with polyethylene glycol has shown flake-like morphology, which fragments above 700 °C,^52^ consistent with the features observed in the BCO samples. SEM-EDS mapping and spectra (Figures S2 and S3) confirmed the presence of Bi, Co, and O as the main elements, heterogeneously distributed within the sillenite, Co_3_O_4_ and Bi_2_O_3_ composite oxides.

**Figure 3:**
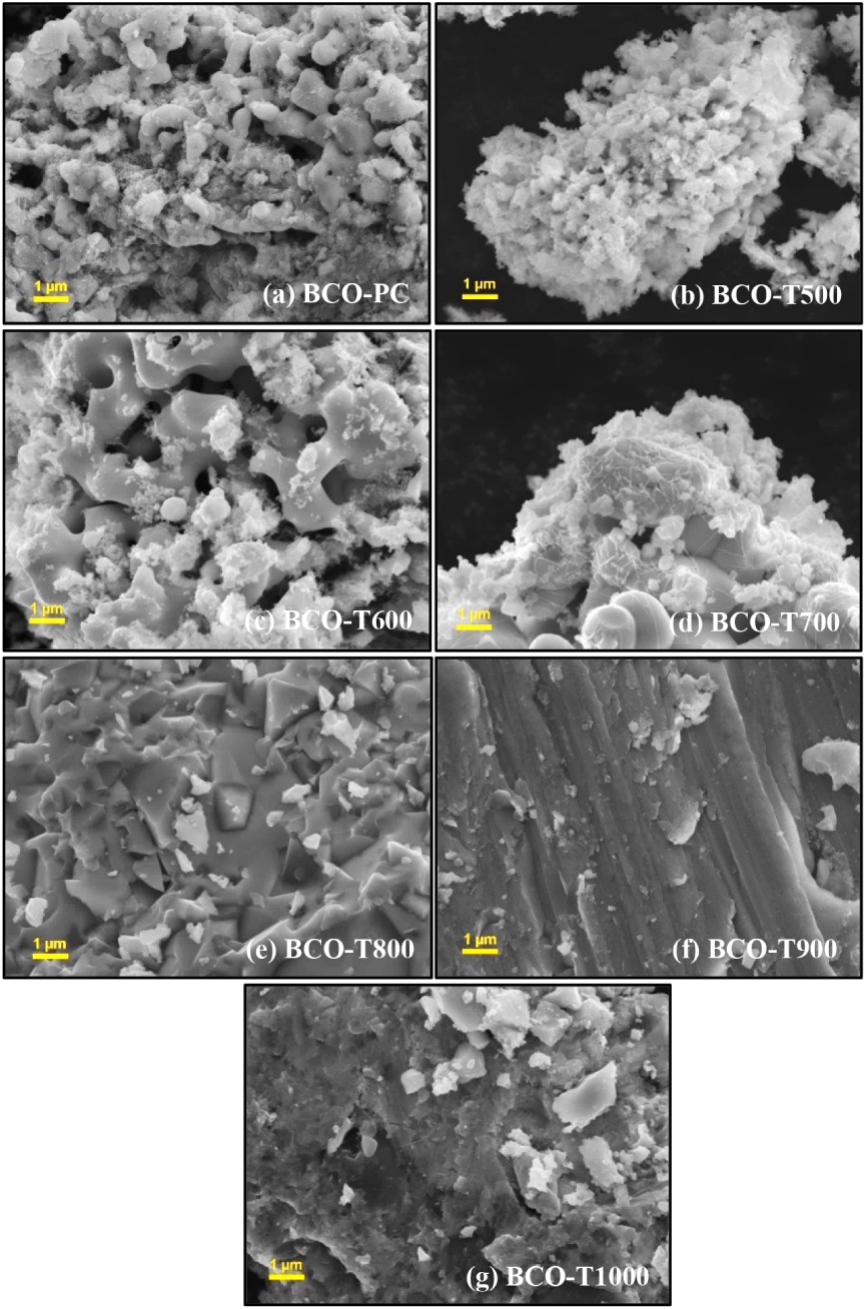
FESEM micrographs at 10000× magnification: (a) BCO-PC, (b) BCO-T500, (c) BCO-T600, (d) BCO-T700, (e) BCO-T800, (f) BCO-T900, and (g) BCO-T1000.

The TEM micrographs of BCO-T700 (Figure 4) reveal agglomerates formed by the coalescence of the primary particles into larger secondary structures. High-magnification analysis shows predominantly cuboid morphologies, along with some irregular particles, with an average size of ∼ 141 nm. The crystalline nature of BCO-T700 was confirmed by selected area electron diffraction (SAED). The planes observed in the SAED pattern correspond to reflections at 2θ ≈ 30° and 36°, associated with the (222) and (330) crystallographic planes of the sillenite phase. These assignments are in agreement with the XRD results. Scanning transmission electron microscopy (STEM)-EDS mapping further demonstrated a heterogeneous distribution of Bi, Co, and O. The formation of these nanoscale particles can be attributed to the efficiency of the Pechini synthesis method. Similar observations have been reported in the literature. Madji et al. described uniform cubic grains for Bi_12_CoO_20_ sillenites,^53^ while Muthu Kumar et al. observed that Bi_25_FeO_40_ sillenites formed cubic structures composed of finer nanoparticles, which exhibited high surface area and exceptionally accessible active sites.^54^

**Figure 4:**
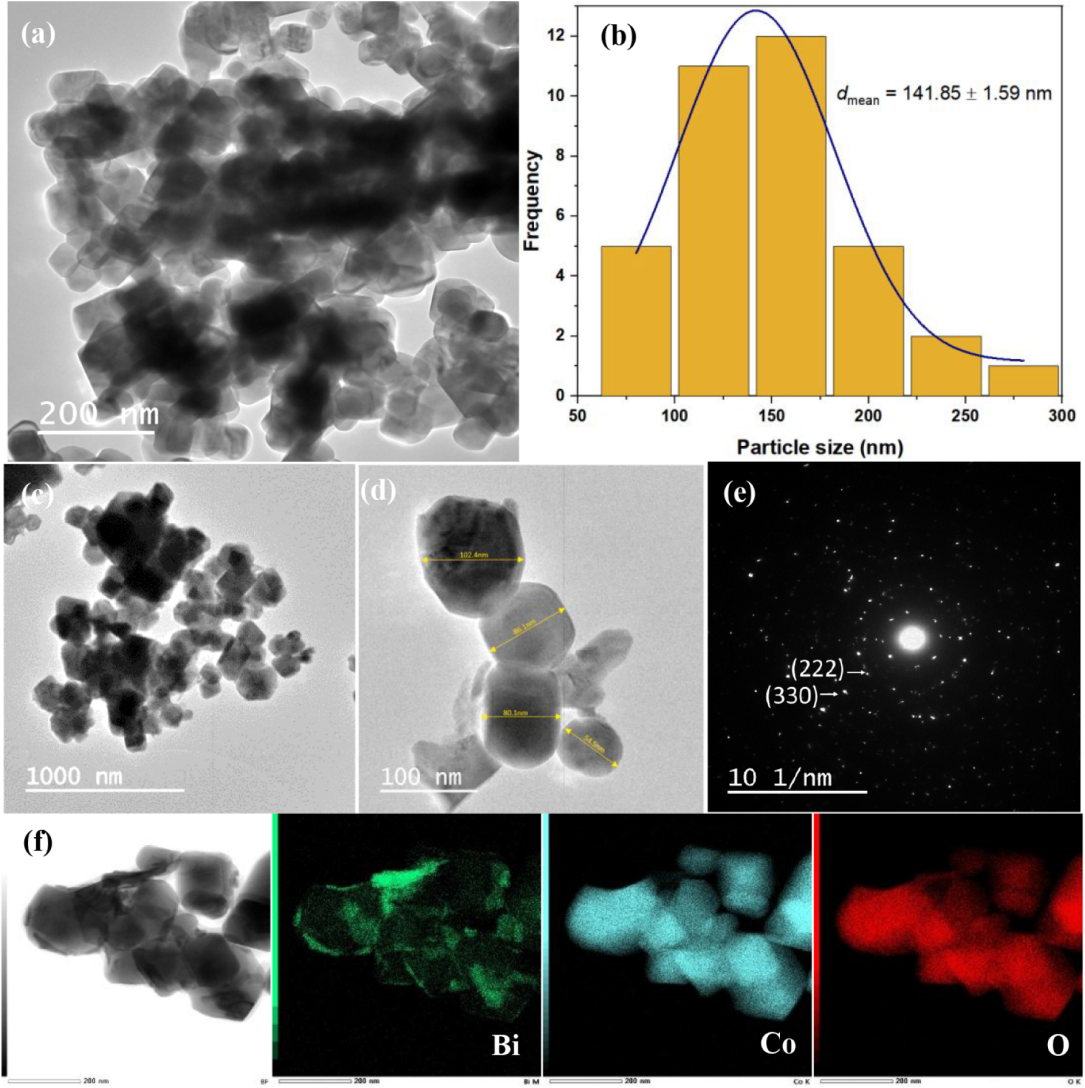
(a, c, d) TEM micrographs of BCO-T700; (b) histograms of nanoparticle size distribution of image (a); (e) SAED; and (f) STEM-EDS elemental mapping of Bi, Co, and O.

More information regarding the chemical composition is derived from XPS analysis. The survey spectra of both precalcined and calcined BCO samples are shown in Figure S4. In all cases, the characteristic Co 2p_3/2_, O 1s, and Bi 4f_7/2_ fingerprints are observed at 780.7, 530.4, and 159.4 eV, respectively. No impurities beyond carbon were detected. Details of the oxidation states are provided through the high-resolution spectra displayed in Figure 5. Overall, the Co 2p (top panel) is mainly composed by Co^2+^ and Co^3+^ states, at 781.5 ± 0.8 eV and 779.4 ± 0.3 eV, respectively, with an additional cobalt hydroxide component at 783.2 ± 0.8 eV, in agreement with previous results.^55,56^ In the O1 spectra (middle panel), besides oxygen lattice (O I) at 529 ± 0.4 eV, hydroxyl groups (O II) at 531 ± 0.6 eV are also noted. In addition, oxygen in a dative-bond configuration (O III),^57^ and water and/or carbonate species (O IV)^58^ are found at 532 ± 0.7 and 534 ± 0.5 eV, respectively. The Bi 4f spectra reveal the existence of bismuth­(III) hydroxide species, as depicted in Figure 5 (bottom panels). The components at 157.8 ± 0.3 eV and 163.3 ± 0.3 eV correspond to the Bi 4f_7/2_ and Bi 4f_5/2_ in the +3 configuration,^59^ whereas the components at 160.3 ± 0.4 eV and 165.7 ± 0.4 eV reveal the presence of bismuth­(III) hydroxide. Details regarding the oxidation state of cobalt and bismuth species will be connected next after the presentation of electrochemical results.

**Figure 5:**
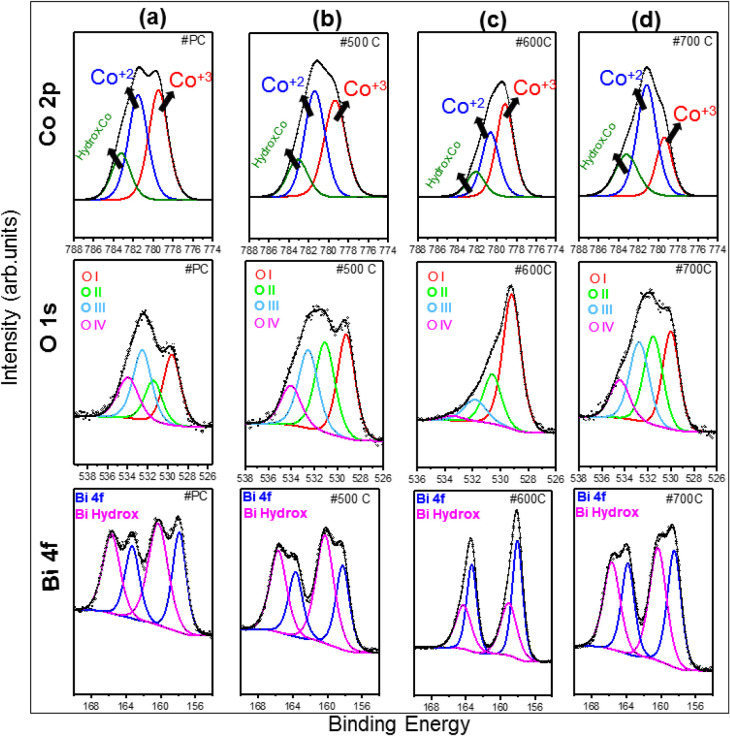
XPS analysis of BCO-PC (a), BCO-T500 (b), (c) BCO-T600, and (d) BCO-T700. The main peaks, Co 2p_3/2_ (top panel), O 1s (middle panel), and Bi 4f (bottom panel), are shown. The Co 2p background was subtracted for better contrast between the components.

The FTIR spectra of the Bi and Co oxides observed in Figure S5 are dominated by the characteristic sillenite bands below 600 cm^–1^, with peaks nearly 462 and 523 cm^–1^ assigned to asymmetric axial deformations of Bi–O bonds in [BiO_7_] units,^54^ and the band at 579 cm^–1^ attributed to Bi–O–Co stretching in the sillenite phase.^12,15^ The 665 cm^–1^ band is assigned to Co–O vibrations in Co_3_O_4_, which is dependent on Co content in the lattice.^12,15^ The bands observed in the range 1400 – 800 cm^–1^ are assigned to carbonate groups (CO_3_^2–^), which usually arises from EG thermal decomposition,^60^ although the band at 846 cm^–1^ associated with EG disappears after 500 °C. Additional features include a band at 1270 cm^–1^ (C–C­(=O)–O stretching from the esterification of CA),^61,62^ bands at 1388 and 1454 cm^–1^ (N–O stretching of NO_3_^–^ complexes),^12,15^ a 1636 cm^–1^ band (H–O–H bending of adsorbed water),^63^ and a 1722 cm^–1^ band (carboxylate C=O stretching),^61^ whose intensity decreases with increasing calcination temperature. Bands between 1270 and 1722 cm^–1^, typically observed in sol–gel derived materials, are attributed to residual organics from gelation.^12,15^ A summary of the main mixed oxide bands is given in Table S4.

The Raman spectra of the Bi and Co composite oxides are shown in Figure 6 and exhibit a set of characteristic bands attributed to different vibrational modes of the crystal lattice. The band at 148 cm^–1^ is related to the coupled breathing of bismuth and oxygen atoms, while the peak at 197 cm^–1^ corresponds to Bi–O bond vibrations.^12,64^ At higher frequencies, the band at 474 cm^–1^ is associated with CoO_4_ units in tetrahedral sites of the sillenite, and a nearby mode at 488 cm^–1^ is characteristic of the Co_3_O_4_ phase.^65^ Another significant contribution from the sillenites is observed at 526 cm^–1^, assigned to breathing involving only oxygen atoms in the Bi–O lattice.^53,66^ The band at 624 cm^–1^ is related to weak Bi–O vibrations, whereas the region at 694 cm^–1^ corresponds to the Co_3_O_4_ phase.^65^ A summary of the main Raman features is provided in Table S5. In the BCO-PC to BCO-T900 materials, mainly sillenite and two prominent Co_3_O_4_ bands are observed. Furthermore, increasing the calcination temperature to 1000 °C promotes the predominance of the Bi_2_O_3_ phase, as confirmed by the enhanced Raman bands at 124, 315, and 453 cm^–1^, corroborated by XRD analysis of BCO-T1000.^67,68^ For the Raman mapping analysis (Figure S6), the colors were assigned to the bands at 148, 197, and 694 cm^–1^, to highlight variations in signal intensity across the sample surface, where warmer colors indicate areas with higher Raman intensity, while cooler colors represent lower intensity. Across all samples, the Raman mapping results reveal a predominantly homogeneous spatial distribution of the selected vibrational modes. Although slight local intensity variations are observed, particularly in samples subjected to higher calcination temperatures, the overall phase distribution remains continuous and uniform throughout the mapped areas.

**Figure 6:**
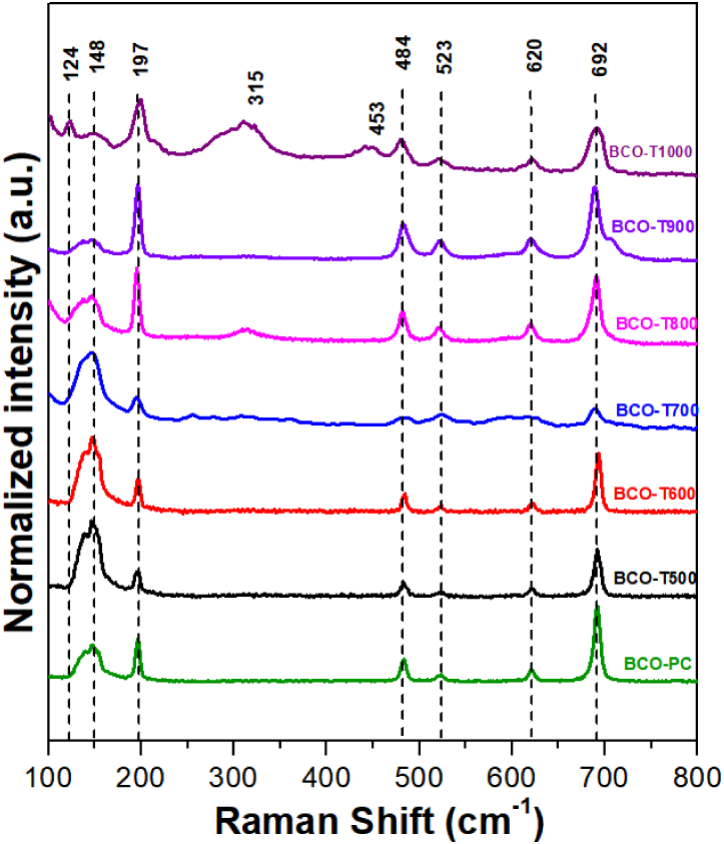
Raman spectra of the BCO composites.

### 3.2 Electrochemical Characterization

The electrocatalytic performance of the Co-based sillenite films toward the OER and HER was initially assessed by LSV. Subsequently, a comprehensive electrochemical analysis was carried out, including the evaluation of the electrochemical double-layer capacitance, charge transfer resistance, electrochemically active surface area, specific activity, Tafel slope, overpotential, and Faradaic efficiency, using data obtained from CV, EIS, LSV, and stability studies by chronopotentiometry. All the results obtained for the BCO materials calcined in the range of 300 – 700 °C and commercial RuO_2_ are gathered in Table 2.

**Table 2:** Electrochemical Data for OER and HER at pH 14 Catalyzed by the BCO Samples^a^ ^b^

Catalysts	**η**_**OER**_ **(mV)**	**η**_**HER**_ **(mV)**	**Tafel**_**OER**_ **(mV dec** ^**–1**^ **)**	**Tafel**_**HER**_ **(mV dec** ^**–1**^ **)**	**ECSA** **(cm** ^**2**^ **)**	**RF**	***R***_**ct total**_ **(kΩ)**	**SA**_**OER**_ **(mA cm** ^**–2**^ **)**	**SA**_**HER**_ **(mA cm** ^**–2**^ **)**
BCO-PC	521	323	167	69	0.657	3.35	1.69	0.869	1.45
BCO-T500	553	326	168	97	0.771	3.93	0.681	0.302	1.56
BCO-T600	486	323	62	76	0.324	1.65	1.09	1.58	2.87
BCO-T700	475	307	75	23	0.116	0.593	1.81	8.44	11.5

a Overpotential η_OER_ at *j* = 10 mA cm^–2^, η_HER_ at *j* = −10 mA cm^–2^, SA_OER_ at η = 450 mV, and SA_HER_ at η = 300 mV.

b RuO_2_: η_OER_ = 371 mV, η_HER_ = 136 mV, Tafel_OER_ = 96 mV dec^–1^, Tafel_HER_ = 65 mV dec^–1^.

The CV of the sillenite-based composites shown in Figure 7 revealed multiple redox peaks, associated with the complex multicationic composition and atomic arrangements of these materials. The presence of Bi and Co cations favors the occurrence of multiple redox processes, commonly leading to distinct anodic and cathodic peaks.^54^ These processes are quasi-reversible at a scan rate of 100 mV s^–1^ and are consistent with the Bi^0^/Bi^3+^ and Co^2+^/Co^3+^ redox couples reported for Bi_2_O_3_^69^ and sillenites.^54,70^ Such features are strongly influenced by both the chemical composition and crystalline structure of the materials. Interestingly, the current density of the redox peaks decreases with the calcination temperature, indicating a decrease in the accessibility of the electrochemically active sites, which was further supported by the ECSA data discussed bellow.

**Figure 7:**
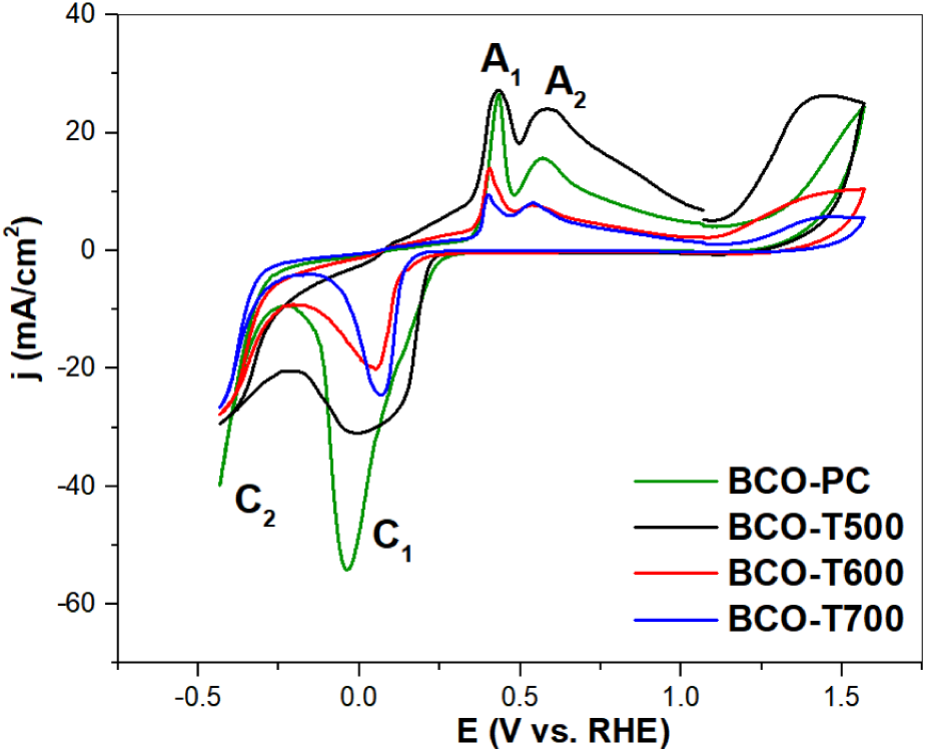
Cyclic voltammograms of BCO composites in 1 mol L^–1^ KOH, using a static GC WE at a scan rate of 100 mV s^–1^.

Anodic peaks A_1_ and A_2_ were observed at approximately 0.40 and 0.55 V vs RHE, respectively. The peak A_2_ is attributed to the oxidation of metallic bismuth species to Bi^3+^,^70^ through the intermediate formation of Bi^+^ (eqs 12–15),^69^ followed by hydroxide interactions and the formation of Bi­(OH)_3_ and BiOOH (eqs 14 and 15),^69^ while peak A_1_ is likely related to the oxidation of a smaller population of Bi^0^ species located near the metal–electrolyte interface. Although it involves the same redox mechanism described for A_2_, the distinct location and accessibility of these surface Bi atoms result in a separate anodic signal.

12 $${\mathrm{Bi}}_{\mathrm{metallic}}\to {\mathrm{Bi}}^{+}{+e}^{-}$$

13 $${\mathrm{3Bi}}^{+}⇌{\mathrm{Bi}}^{3+}{+\mathrm{2Bi}}_{\mathrm{metallic}}$$

14 $${\mathrm{3OH}}^{-}{+\mathrm{Bi}}^{3+}\to \mathrm{Bi}{\left(\mathrm{OH}\right)}_{3}$$

15 $$\mathrm{Bi}{\left(\mathrm{OH}\right)}_{3}\to {\mathrm{BiOOH}+H}_{2}O$$

The cathodic peaks C_1_ (≈0 V vs RHE) and C_2_ (≈−0.4 V vs RHE) were assigned to the stepwise reduction of BiO_2_^–^ species. C_1_ may correspond to the reduction of adsorbed BiO_2_^–^ to Bi^0^ (eqs 16–19),^69^ while C_2_ reflects the reduction of dissolved BiO_2_^–^ species (eqs 20–22),^69^ as proposed in previous studies. The presence of multiple cathodic peaks suggests disproportionation of BiO_2_^–^ intermediates, and the appearance of additional redox signals may be due to catalytic induction or incomplete oxidation of Bi^0^, a phenomenon also reported in other Bi-based mixed oxides.^71–73^

16 $${\mathrm{Bi}}_{2}O_{3}{+\mathrm{2OH}}^{-}\to {{\mathrm{2 BiO}}_{2}}^{-}{+H}_{2}O$$

17 $${{\mathrm{BiO}}_{2}}^{-}{\mathrm{+ e}}^{-}\to {{{\mathrm{BiO}}_{2}}^{\mathrm{2-}}}_{\mathrm{ads}}$$

18 $${2H}_{2}O+3{{{\mathrm{BiO}}_{2}}^{\mathrm{2-}}}_{\mathrm{ads}}⇌2{{{\mathrm{BiO}}_{2}}^{-}}_{\mathrm{ads}}+4OH^{-}+{{\mathrm{Bi}}^{0}}_{\mathrm{ads}}$$

19 $${{\mathrm{Bi}}^{0}}_{\mathrm{ads}}\to {\mathrm{Bi}}_{\mathrm{metallic}}$$

20 $${{\mathrm{BiO}}_{2}}^{-}{+e}^{-}\to {{\mathrm{BiO}}_{2}}^{2-}$$

21 $${2H}_{2}O+3{{\mathrm{BiO}}_{2}}^{2-}⇌{{\mathrm{2BiO}}_{2}}^{-}{+\mathrm{4OH}}^{-}{+\mathrm{Bi}}^{0}$$

22 $${\mathrm{Bi}}^{0}\to {\mathrm{Bi}}_{\mathrm{metallic}}$$

EIS characterization was carried out to evaluate the total charge transfer resistance for the BCO samples. The Nyquist Plots (Figure 8) confirmed the expected trend: lower calcination temperatures led to lower *R*_ct_ values (Table 2), indicating improved charge transfer kinetics, except for BCO-PC, which is probably more resistant because of the residual organic CA and EG. Moreover, the BCO materials exhibited relatively low *R*_ct_ values, for instance, BCO-T700, R_ct_ of 1.81 kΩ was obtained, which is lower than the resistances of sillenite samples reported in the literature, such as the 31.8 kΩ found for Bi_12_CoO_20_ by Kenfoud et al.^73^ The EIS data also revealed low solution resistance for BCO (*R*_*s*_: 9.5–14.5 Ω) and the absence of low-frequency diffusion tails, indicating minimal ionic diffusion limitations. These characteristics further support the promising electrocatalytic performance of the BCO catalysts. The effective capacitance is shown in Table S6.

**Figure 8:**
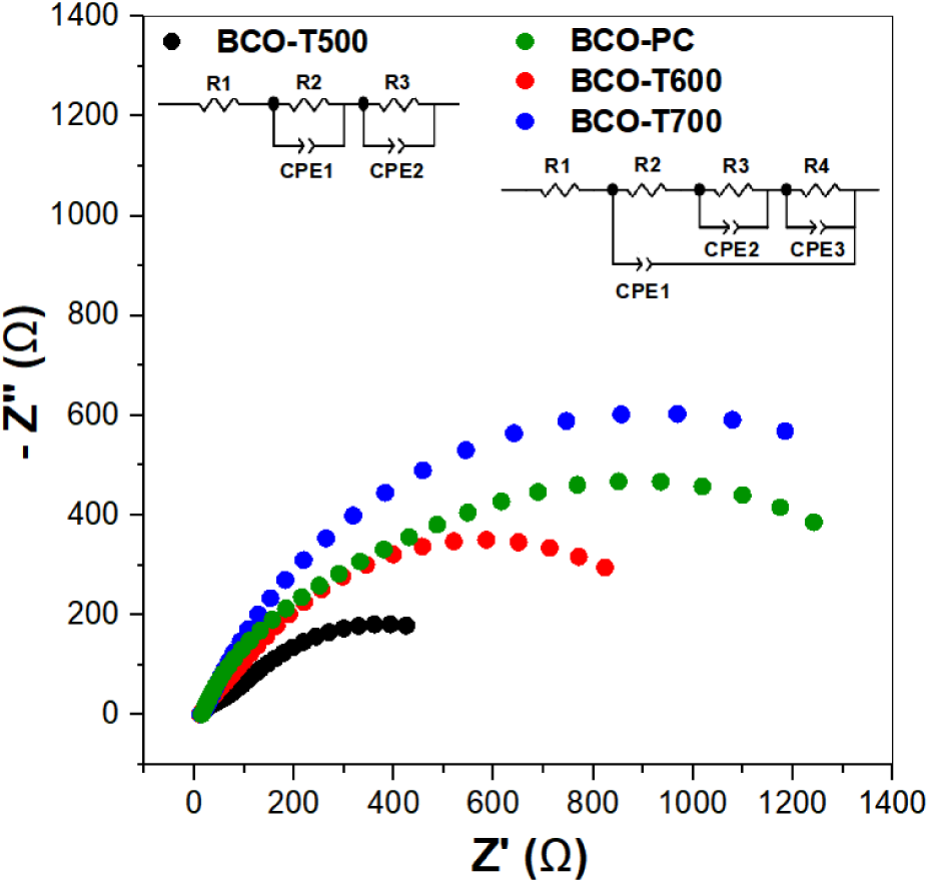
EIS of BCO composites in 1 mol L^–1^ KOH FTO WE: Nyquist diagram and the respective circuits.

Another approach to represent the EIS data and to complement the information regarding the behavior of electrochemical systems is the Bode plot (Figure S7), which displays the phase angle as a function of the logarithm of frequency.^74^ In ideal systems, a purely capacitive response results in a phase angle close to 90°, whereas a purely resistive system exhibits values near 0°.^75^ However, transition metal oxides typically show intermediate behavior between 40° and 70°, characteristic of a constant phase element (CPE), arising from surface heterogeneity, porous structures, or the presence of multiple simultaneous electrochemical processes. The porosity and roughness of the electrode surface are expected to induce frequency dispersion in the interfacial impedance, even without considering the more complex current-distribution effects.^76^ The BCO-T700 sample exhibits a response close to that of an ideal capacitor, along with a phase angle shift (57°) toward lower frequencies, indicating a higher R_ct_. In contrast, the BCO-T600 (45° and 47°) and BCO-T500 (29° and 34°) samples display two distinct phase maxima, which may be attributed to kinetic effects at high frequencies and mass transfer effects at low frequencies.^75,76^

### 3.3 Water-Splitting Electrocatalysis

The LSV curves under OER and HER conditions, obtained for the sillenite-based electrocatalysts calcined up to 700 °C, are shown in Figure 9. Electrocatalytic metrics are shown in Table 2. For the samples BCO-T800, BCO-T900, and BCO-T1000, low-quality films were obtained, probably associated with the sintering of the samples at higher thermal treatment, leading to high overpotentials, as shown in Figure S8. Benchmark RuO_2_ OER and HER assays are shown in Figure S9 for comparison.

**Figure 9:**
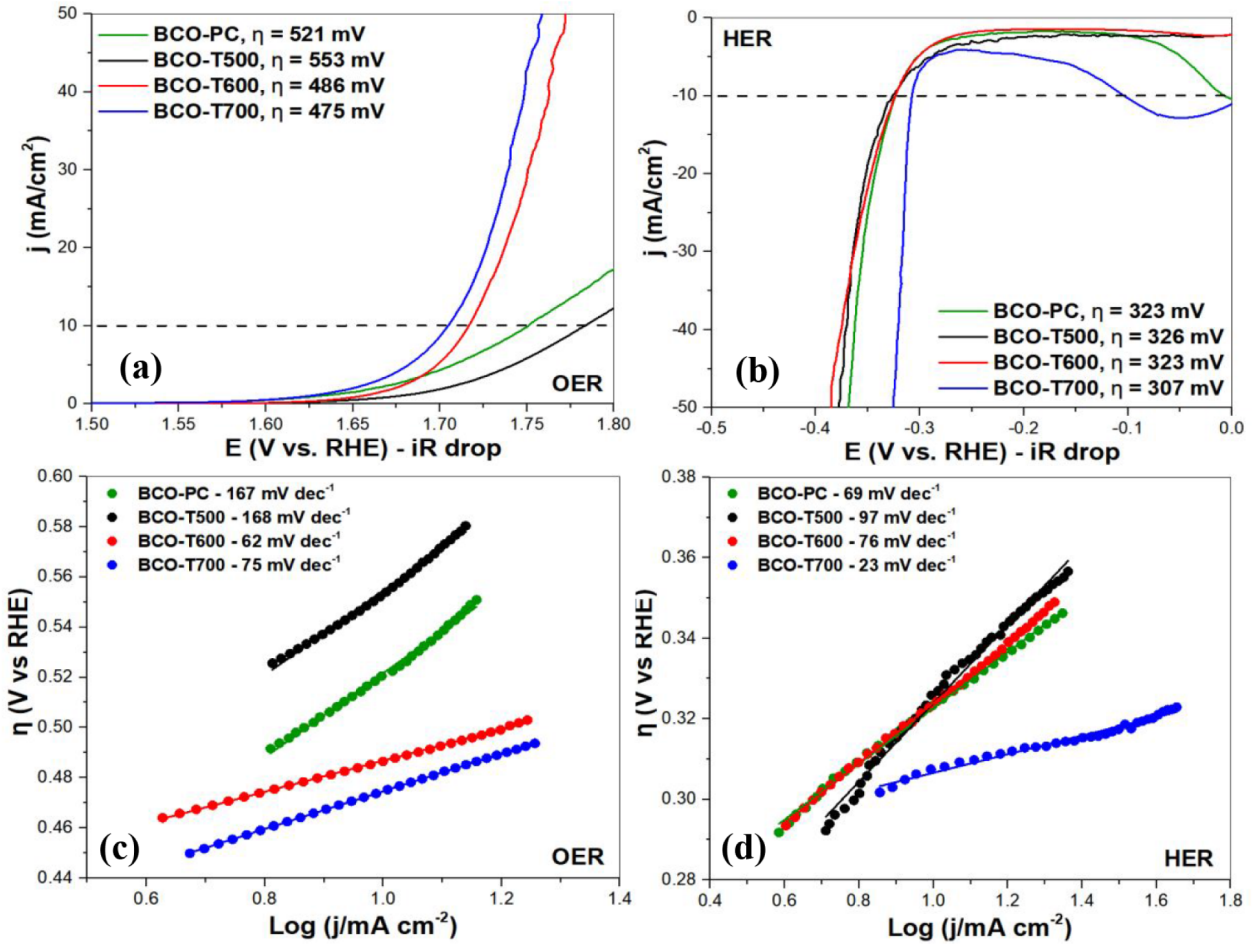
OER and HER activity for the BCO samples in 1 mol L^–1^ KOH with CG RDE WE. Linear sweep voltammograms with 85% *iR* compensation for (a) the OER and (b) the HER; Tafel plots for (c) the OER and (d) the HER.

For the OER, the overpotential at 10 mA cm^–2^ decreases with the raising of the calcination temperature; the BCO-T700 sample delivered the lowest value (475 mV), suggesting that a higher sillenite fraction may favor water oxidation. In contrast, BCO-T500 exhibited a higher overpotential in the OER (553 mV), likely due to its reduced sillenite content and increased α-Bi_2_O_3_ fraction. At higher current densities, the overpotential of BCO-T600 and BCO-T700 became considerably lower in comparison with BCO-PC and BCO-T500. In relation to the BCO-PC, it was not possible to refine the XR diffractogram due to the poor crystallinity of the sample, but the lower overpotential achieved in relation to BCO-T500 (521 mV) can be associated with the amorphous nature of the oxide and defect-rich structure that may favor electrocatalysis. These findings reinforce the idea that the ethylene glycol-to-citric acid ratio plays a role in modulating electrocatalytic performance across all samples, possibly through structural factors such as phase composition, crystallinity, defect distribution, morphological control, or charge transport. Although Co_3_O_4_ is widely recognized for its high activity in water oxidation, its relatively constant content across the samples (30–32%) precludes direct correlation with overpotential variations. Nevertheless, a synergistic effect among Co_3_O_4_, the sillenite phase, and Bi_2_O_3_ is likely contributing to the overall catalytic behavior.

The electrocatalytic kinetics of the OER were further investigated by Tafel plots, as shown in Figure 9d. The Tafel slope, obtained from the linear region of the η vs log j plot, is a fundamental parameter that reflects the rate-determining step in an electrochemical reaction.^39^ In general, smaller Tafel slopes are indicative of more favorable reaction kinetics. The BCO-T600 and BCO-T700 samples exhibited the lowest Tafel slopes (respectively, 62 and 75 mV dec^–1^), consistent with their low overpotentials and enhanced electrocatalytic activity. In contrast, BCO-PC and BCO-T500 displayed higher slopes, suggesting slower charge-transfer processes during the OER.

For the HER, the lowest overpotential (307 mV at 10 mA cm^–2^) was also achieved by BCO-T700, although the HER performance among the samples was less dependent on phase composition and heat treatment than for the OER. The BCO-T700 sample achieved the lowest Tafel slope (27 mV dec^–1^), which indicates superior reaction kinetics compared to the other samples. These results are in good agreement with the overpotential trends discussed previously for OER and reinforce the kinetic advantage of the BCO-T700 sample for both OER and HER in alkaline media.

Notably, BCO-T700 exhibited not only the lowest overpotentials for the OER and HER but also the highest specific activity for both reactions, indicating a superior intrinsic catalytic performance that can be associated with the highest content of the sillenite phase in this sample. The SA values were obtained by normalizing the catalytic current at a fixed overpotential by the ECSA (Figures S10 and S11), thus reflecting the intrinsic activity of the electrochemically accessible sites.^77^ This high SA compensates for its lower ECSA and roughness factor, which indicates that this mixed oxide possesses a higher intrinsic activity per site/area, likely arising from its major sillenite phase composition. It is considered that ECSA can underestimate the density of truly active sites in mixed oxides due to additional oxide-layer capacitance and pseudocapacitive or faradaic contributions from semiconductive surfaces.^78^ These factors can artificially inflate or degrade the apparent capacitance, obscuring its direct relation to the actual catalytic interface. Figure 10 shows the correlation between the phase composition and the overpotential and specific activity for both the OER and HER. It is evident that the increase in the sillenite phase content across the materials led to enhanced water-splitting performance.

**Figure 10:**
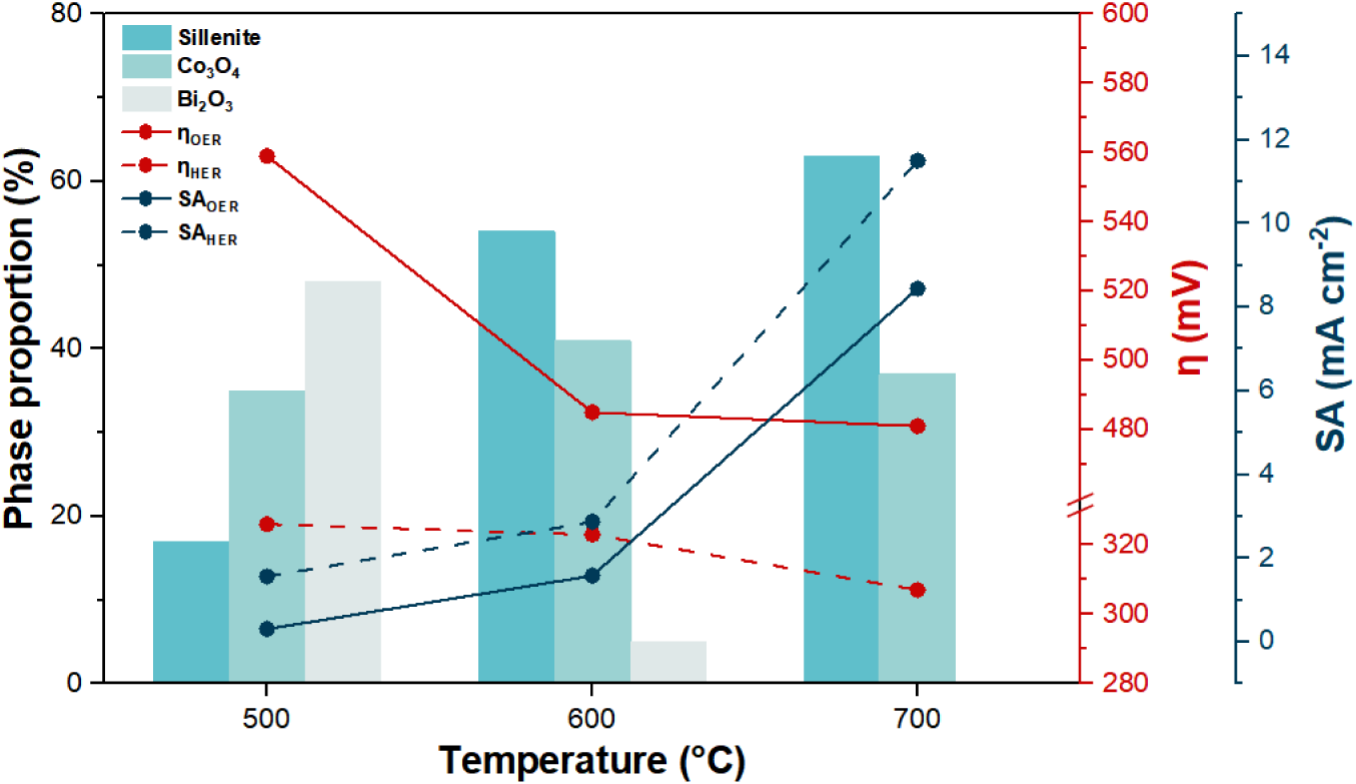
A comparison of the phase composition in relation to overpotential and specific activity in the OER and HER. Solid lines: OER; dashed lines: HER.

To understand the relation between the sillenite phase and the electrocatalytic OER/HER activity, a detailed analysis of the XPS data provided information about the effect of the surface composition of the samples on the electrocatalytic activity. When comparing the signatures of the precalcined samples with those of the calcined samples (Figure 5a–d), slight yet important modifications emerge. For instance, for BCO-PC, the Co^2+^ and Co^3+^ contributions are nearly identical, resulting in a Co^2+^/Co^3+^ ratio close to one. In contrast, the bismuth­(III) hydroxide displays a higher contribution than the Bi^3+^ signal, as noted in Figure 5a. The sample BCO-T500 displays a prevalence of Co^2+^, as shown in Figure 5b (top panel). The increased intensity of hydroxyl oxygen (O II), shown in Figure 5b (middle panel), correlates with the higher fraction of bismuth (III) hydroxide observed in the Bi 4f envelope Figure 5b (bottom panel). Although the Co^2+^ dominancy seems promising for hydrogen and/or oxygen reactions, the synergistic prevalence of Bi^3+^ states is a requirement for catalytic feasibility. Furthermore, bismuth sites stabilize surface hydroxyl groups and modulate the local electronic structure around cobalt, thereby improving charge transfer during HER and OER.^59^ In addition, excessive hydroxylation leads to partial surface blocking and reduced electronic conductivity, limiting the catalytic performance. Therefore, the sample calcined at 500 °C is not ideal for such processes and actually reaches the highest overpotentials for the OER and HER, as discussed ahead. Likewise, the BCO-T600 is not the most suitable for hydrogen and/or oxygen evolution reactions either , as revealed in Figure 5c. Notably, the Co^2+^/Co^3+^ ratio is below 1, indicating that cobalt predominantly arises in the +3 state, which tends to decrease the reaction capability. Although the sample displayed lower hydroxide contributions, highlighted by the decrease in the number of O II species (Figure 5c middle panel) and lower bismuth hydroxide content (Figure 5c bottom panel), resulting in a higher Bi^3+^ concentration, the absence of Co^2+^/Bi^3+^ synergy still limits the overall catalytic capability. On the other hand, the sample calcined at 700 °C reveals a well-balanced distribution of Co^2+^ and Bi^3+^ species, as indicated in Figure 5d. The Co 2p envelope is dominated by Co^2+^ states (Figure 5d top panel), yielding a Co^2+^/Co^3+^ ratio of approximately 2.3. Although the O 1s spectrum does not reveal an appreciable decrease in the O II component (Figure 5d middle panel), the bismuth hydroxide contribution seems lower than that of Bi^3+^ (Figure 5d bottom panel). Such a synergistic combination of Co^2+^ and Bi^3+^ explains the superior HER and OER performance of the BCO-T700 sample.

The composite material BCO-T700 exhibited an OER overpotential within the range reported for bismuth–iron sillenites (Table S7), showing lower values than those described by Vijay et al. (640 mV/1 mA cm^–2^)^10^ and comparable to those obtained by Arora et al. (420 mV/10 mA cm^–2^).^7^ In contrast, the HER overpotentials of BCO-T700 were higher than those reported in the literature for Co_3_O_4_- and Bi_2_O_3_-based materials. For instance, Co_3_O_4_ synthesized by Liu et al. reached 195 mV/–10 mA cm^–2^,^79^ while Bi_2_O_3_ supported on metallic Bi and Ni foam achieved 250 mV/–10 mA cm^–2^.^39^ Nevertheless, to the best of our knowledge, this study provides the first report on the application of sillenites toward the HER.

Stability tests under OER conditions were performed by chronopotentiometry over a period of 3 h at a current density of 10 mA cm^–2^, using an FTO WE. As illustrated in Figure S12, the BCO-T500 film exhibited the lowest overpotential, 820 mV after 3 h, which motivated its selection for extended durability analysis. The long-term stability of BCO-T500 over 18 h showed a continuous but slight increase in overpotential throughout the stability test, reaching 1070 mV and evidencing a gradual loss of activity. However, the BCO-T500 film showed the lowest Faradaic efficiency during the 3-h evaluation. In contrast, BCO-T700 presented the lowest stability, reaching an overpotential of 1325 mV at 2 h and the higher Faradaic efficiency of 25%. Comparative analysis revealed that the stability of the films followed the trend BCO-T500 > BCO-T600> BCO-PC > BCO-T700, whereas the Faradaic efficiency exhibited a different order, BCO-T700 > BCO-PC > BCO-T500 > BCO-T600, with respective values of 25%, 13%, 11%, and 9% (Figures S13 and S14).

Stability tests under HER conditions were performed at – 5 mA cm^–2^ for 2 h, as shown in Figure S15. At higher currents, the film detached from the FTO substrate immediately. All materials showed similar stability, starting with an overpotential around 350 mV and reaching 450 mV after 2 h; however, detachment of the FTO was observed after this period. The Faradaic efficiency followed the order BCO-T700 > BCO-T600 > BCO-T500 > BCO-PC, with the respective values of 22%, 18%, 14%, 11%.

To investigate the origin of the limited stability and low Faradaic efficiency of the materials toward both the OER and HER, as well as other possible reactions taking place at the working electrode, characterization of the FTO film was carried out after chronopotentiometry.

The XRD of the spent films is characterized by diffraction planes of the FTO substrate and the original crystalline phases, as well as new phases formed during the chronopotentiometry under the OER and HER conditions. It is noteworthy that the interference of the FTO peaks and a possible preferred orientation during the formation of the films prevent unequivocal assignment of the phases. The post-OER diffractograms (Figure S16) of BCO-PC, BCO-T500, and BCO-T600 indicate the formation of the perovskite BiCoO_3_ (cubic), while for BCO-T700, CoO_2_ was suggested. XRD after HER (Figure S17) indicates the formation of Co­(OH)_2_ and Bi for BCO-PC and BCO-T500, and CoO­(OH), CoO_2_ and Bi for BCO-T600 and BCO-T700. The formation of Bi(0) was also evidenced by CV in the cathodic scan. These phase transformations at the electrode during prolonged chronopotentiometry consume part of the applied current, decreasing the charge delivered to oxidize the water and, consequently, the Faradaic efficiency discussed above.

XPS analyses were carried out for BCO-T700 after HER and OER stability, since this sample presented the lower overpotential. In this regard, Figure 11 displays the XPS main lines’ high-resolution spectra. Pronounced modifications in BCO T700 were observed after the OER experiments, as depicted in Figure 11a–c. The cobalt oxidation state shifts from a Co^2+^-rich surface before the OER to a Co^3+^-rich one, as shown in Figure 11a. This transition suggests that Co^2+^ is highly consumed throughout the reaction, decreasing the Co^2+^/Co^3+^ ratio to 0.67. A new component at a high binding energy around 795 eV is observed, suggesting further hydroxylation of BCO. This finding agrees with the rise of the O II component in the O 1s envelope, as noted in Figure 11b. In addition to the decrease of the O I component, it can be observed that dative oxygen and water/carbonate species decrease (O III) or even vanish (O IV). These findings suggest metal oxide species are consumed and that the sample becomes more homogeneous than before the reaction, in the sense that fewer impurities remain (the O IV contribution becomes negligible), and more impurity-free Bi–O–Co chains (decrease of O III components) result. The sharp, shoulder-less Bi 4f spectra shown in Figure 11c reinforce these hypotheses. While the Bi 4f_7/2_ required two components for fitting the envelope, assigned to Bi^3+^ in an oxide-like environment at 157.9 eV and Bi­(III) hydroxide species at ∼ 159.8 eV, the Bi 4f envelope collapses into a single, sharper doublet centered at ∼158.9 eV after the OER experiments. At first glance, one might suspect that this peak represents either a unique Bi^3+^ or Bi–O­(H) signature. However, this interpretation would require assuming an overall binding energy shift of roughly 1 eV relative to the prereaction state. Although the prevalence of Co^3+^ species after the reaction may suggest a more conductive environment, such an overall shift is not observed in the other spectral regions, making this explanation less likely. One could also suspect that all Bi^3+^ species were fully consumed, leaving only Bi–O­(H) species. Yet, the O 1s analysis still suggests a bismuth­(III) oxide contribution. Given these constraints, and based on the aforementioned changes in the O 1s signatures, we attribute the post-OER Bi 4f feature to Bi^3+^ in a more homogeneous Bi–O­(H) environment. In this scenario, the expected consumption of Co^2+^ and partial restructuring of bismuth­(III) oxide domains lead to stronger final-state screening such that oxide-like Bi^3+^ and hydroxylated bismuth become indistinguishable within the experimental resolution. The shape of Bi 5d states before and after the reactions further highlights the proposed high homogeneity of the BCO-T700 after OER, as shown in Figure S18. While before the reaction the Bi 5d appears broad, shapeless, and lacking a well-defined 5d_5/2_-5d_3/2_ splitting, after the reaction this region becomes sharper, more structured, clearly revealing Bi 5d_5/2_ and Bi 5d_3/2_ signatures. Because the Bi 5d levels lie close to the valence band maximum, their binding energies are relatively rigid, but their line shape is highly sensitive to disorder and final-state screening. Conversely, for substantial structural or electronic rearrangements, such as the proposed surface homogeneity enhancement, they act as sensitive markers of electronic screening, displaying noticeable modifications in their overall line shape.

**Figure 11:**
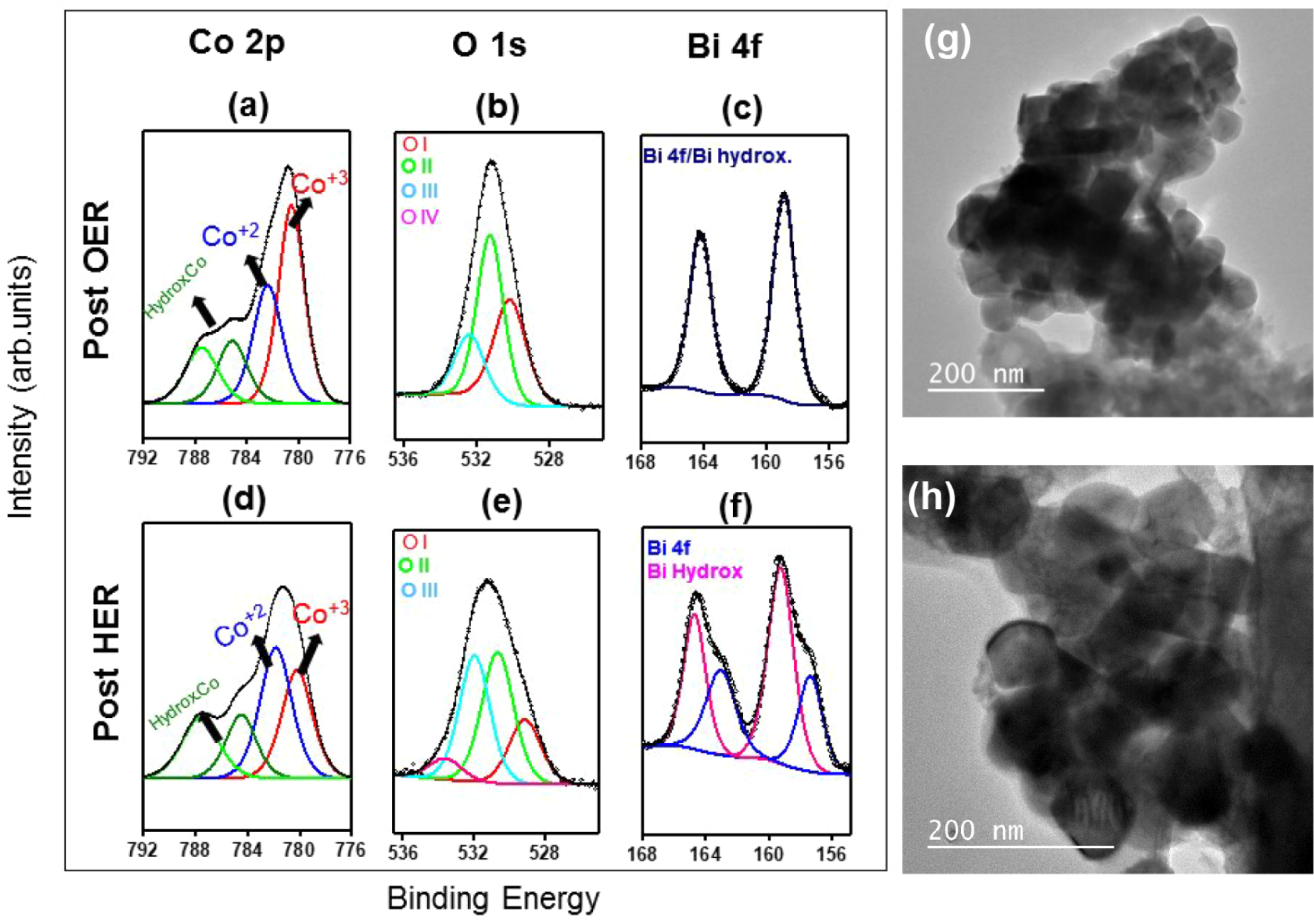
XPS analysis of the BCO-T700 after the (a-c) OER and (d-f) HER (d)-(f) stability tests. The main peaks, Co 2p_3/2_ (left panels), O 1s (middle panels), and Bi 4f (right panel), are shown. The Co 2p background was subtracted for better contrast between the components. High-magnification TEM micrographs of BCO-T700 after OER (g) and HER (h) stability tests.

After the HER process (Figure 11d–f), interesting modifications are observed. On the Co 2p side, the sample remains Co^2+^-rich, yet the Co^2+^/Co^3+^ ratio (1.75) decreases compared to that of the sample before the reaction (2.3), suggesting that part of the Co^2+^ species was consumed in the reaction. In addition, an extra cobalt hydroxide component appears at 790.44 eV, likely derived from hydroxylation during HER. This hypothesis is confirmed through the increase of the hydroxyl component in the O 1s spectra, as revealed in Figure 11e. Furthermore, the contribution of the O I component decreases with respect to the former sample, which suggests that Bi^3+^ is also partially consumed during the reaction. Indeed, as depicted in Figure 11f, the Bi^3+^ states are no longer dominant, decreasing in favor of their hydroxylated bismuth­(III) counterpart. These findings illustrate the importance of the synergetic behavior of Co^2+^ and Bi^3+^ states for the HER process, resulting in the hydroxylation of the BCO catalyst by partially consuming both active sites and causing a partial loss of activity.

TEM micrographs were carried out for BCO-T700. The postreaction images (Figures 11g-h, S19 and S20) revealed localized morphological changes, where some regions exhibited rounding of edges and the development of more spherical or quasi-spherical features. Additionally, a new surface morphology emerged in both films after the electrochemical tests, consistent with the appearance of a new crystalline phase identified in the XRD patterns. Such morphological reconstruction is commonly associated with surface adaptation processes that occur under the OER/HER conditions. TEM-EDS mapping (Figures S19 and S20) confirmed that the heterogeneous distribution of Co, Bi, and O was preserved relative to the pristine material. A minor incorporation of potassium was also detected, originating from the KOH electrolyte used during the stability tests. This incorporation is often reported for transition-metal oxides and does not compromise the catalytic integrity of the films.

After the stability tests, the characteristic Raman bands at 692, 620, 523, 484, 197, and 148 cm^– 1^ remained unchanged for the BCO-PC, BCO-500, BCO-600, and BCO-700 composites under both OER and HER conditions (Figure S21). The full band assignments are listed in Table S5. Comparison of the samples after the OER and HER stability tests with the pristine samples shows a progressive decrease in the intensity of the 148 cm^– 1^ as the intensity of the band at 197 cm^–1^ increases, as the sample preparation temperature increases. This trend suggests that higher-temperature treatments may induce subtle structural rearrangements that selectively weaken this vibrational mode. Moreover, a change in the intensity ratio between the 197 and 148 cm^–1^ bands is more evident post-OER. This difference indicates possible variations in local structural distortions or surface composition that develop preferentially under reducing conditions.

Finally, the overall water splitting was carried out in a two-electrode system in order to evaluate the bifunctional electrocatalytic activity for both the OER and HER. Among the BCO materials, BCO-T500 presented the highest activity. According to the results shown in Figure S22, the material BCO-T500 presented an onset cell potential of 2.36 V, only 290 mV higher than the benchmark catalyst RuO_2_ tested under the same conditions, indicating that the material can be a promising non-noble electrocatalyst for hydrogen production.

## 4 Conclusion

A cost-effective sol–gel Pechini–adapted route was successfully employed to synthesize a series of Co-based sillenite composites containing Co_3_O_4_ and Bi_2_O_3_, whose structural characteristics and electrocatalytic behavior were dependent on the calcination temperature. Structural analyses, including Rietveld refinement, confirmed the coexistence of sillenite phases with slightly different stoichiometries, while morphological characterization revealed diverse micro- and nanoscale morphologies. ICP-OES confirmed Bi:Co ratios near the nominal 1:1 up to 700 °C, evidencing efficient incorporation of the precursors, while higher temperatures led to Co enrichment due to Bi volatilization. Complementary FTIR and Raman spectroscopy further provided insights into the vibrational modes associated with metal–oxygen bonding. Given the limited number of studies on sillenites in electrocatalysis, their application in OER and HER was systematically investigated. Among the samples, BCO-T700 (Bi_18_Co_6_)­Co_2_O_40_–Co_3_O_4_ achieved the lowest overpotential of 475 mV at 10 mA cm^–2^, a Tafel slope of 75 mV dec^–1^, high specific activity of 11.5 mA cm^–2^, and low charge-transfer resistance (1.81 kΩ). Moreover, this study provides the first evidence of sillenite-based composites exhibiting HER activity, with BCO-T700 delivering an overpotential of 307 mV at 10 mA cm^–2^. XPS shows the existence of Co^2+^, Co^3+^, Bi^3+^, and Bi^3+^-hydroxide species, with the synergism between Co^2+^ and Bi^3+^ being directly correlated with the OER and HER activity. The samples exhibited limited stability and low Faradaic efficiency, which was associated with the partial consumption of the applied current to deliver the phase transformation revealed by XRD and XPS analyses after the stability tests. However, a pure sillenite phase would allow a more direct correlation between structure and activity. These findings underscore the potential of Co-based sillenites as versatile, low-cost electrocatalysts for water splitting, reinforcing their relevance in green hydrogen production and paving the way for future applications.

## Supplementary Material



## Notes

Additional characterization data of the BCO materials: ICP-OES, Rietveld refinement, SEM, XPS, FTIR, Raman spectroscopy, EIS, LSV, CV, *C*_*dl*_, chronopotentiometric stability tests, TEM, TEM-EDS, overall water splitting (PDF)
