# Supplementary material for: A Novel Sol–Gel Synthesis Strategy of Co-Based Sillenite Composites for Enhanced Electrocatalysis of Water Splitting
Source: ACS Omega. 2026 Feb 12;11(7):11426–41. doi: 10.1021/acsomega.5c09012 (PMC12947149; doi:10.1021/acsomega.5c09012)
Supplement: Supplementary file 1 [file ao5c09012_si_001.pdf]

A Novel Sol-gel Synthesis Strategy of Co-Based Sillenite Composites for Enhanced Electrocatalysis of Water Splitting

Mayara Acioli dos Santos[a], Leandro Bufaiçal[b], P. R. A de Oliveira[c], Yngrid Synara de Sena Silva[1], Liying Liu[d], Ana Luisa Silva[a], Nakédia M. F. Carvalho[a,*]

[a] Universidade do Estado do Rio de Janeiro, Instituto de Química, Rio de Janeiro, 20550-013, Brazil.

[b] Universidade Federal de Goiás, Instituto de Física, Goiânia, 74690-900, Brazil.

[c] Universidade Federal do Rio de Janeiro, Instituto de Física, Rio de Janeiro, 21941-909, Brazil.

[d] Centro Brasileiro de Pesquisas Físicas, Rio de Janeiro, 22290-180, Brazil.

*Email: nakedia@uerj.br

Summary

S.1. Electrocatalysts characterization…………………………………………….……………S3

S.1.1. Inductively coupled plasma optical emission spectrometry.…………........……S3

S.1.2. X-ray diffraction…………………………………………………........................S4

S.1.3. Scanning electronic microscopy……………………………….........................S5

S.1.4. X-ray photoelectron spectroscopy……………………..………........................S7

S.1.5. Fourier transform infrared spectroscopy………………..…………........……S8

S.1.6. Raman spectroscopy……………………..…………..…………........……S9

S.2. Electrochemical analysis……………………………………………….……………S11

S.2.1. Electrochemical impedance spectroscopy…….………………..………..….....S11

S.2.2. Linear sweep voltammetry..…………………………………..………..….....S12

S.2.3. Cyclic voltammetry……………………………………………..………..….....S13

S.2.4. Chronopotentiometric stability tests………..………………..………..…......S14

S.2.5. Overall water splitting tests ………………..………………..………..…......S21

S.3. OER and HER performance of reported electrocatalysts....…………….……..….....S22

S.4. References……………………………………………………………………..S23

S.1. Electrocatalysts characterization

Table S1. Calcination conditions and nomenclature of the catalyst samples.

Sample	Calcination Temperature (°C)	Nomenclature
1	300	BCO-PC
2	500	BCO-T500
3	600	BCO-T600
4	700	BCO-T700
5	800	BCO-T800
6	900	BCO-T900
7	1000	BCO-T1000

S.1.1. Inductively coupled plasma optical emission spectrometry

Table S2. Elemental quantification, expressed in mg g^{-1}, determined for the BCO composites through digestion with HNO_3 for the analysis of Bi and Co using ICP OES.

Sample	Bi (mg g^{-1})	Co (mg g^{-1})	Bi (mol)	Co (mol)	Formula
BCO-PC	666	173	0.00319	0.00294	$Bi_{1.00}Co_{0.92}O_{2.88}$
BCO-T500	669 ± 10	194 ± 5	0.00320	0.00329	$Bi_{1.00}Co_{1.03}O_{3.04}$
BCO-T600	715 ± 32	205 ± 6	0.00342	0.00348	$Bi_{1.00}Co_{1.02}O_{3.03}$
BCO-T700	728 ± 8	214 ± 4	0.00348	0.00363	$Bi_{1.00}Co_{1.04}O_{3.06}$
BCO-T800	574 ± 12	272 ± 6	0.00275	0.00462	$Bi_{1.00}Co_{1.68}O_{4.02}$
BCO-T900	550 ± 4	301 ± 3	0.00263	0.00511	$Bi_{1.00}Co_{1.94}O_{4.41}$
BCO-T1000	492 ± 13	395 ± 3	0.00235	0.00670	$Bi_{1.00}Co_{2.85}O_{5.78}$

S.1.2. X-ray diffraction

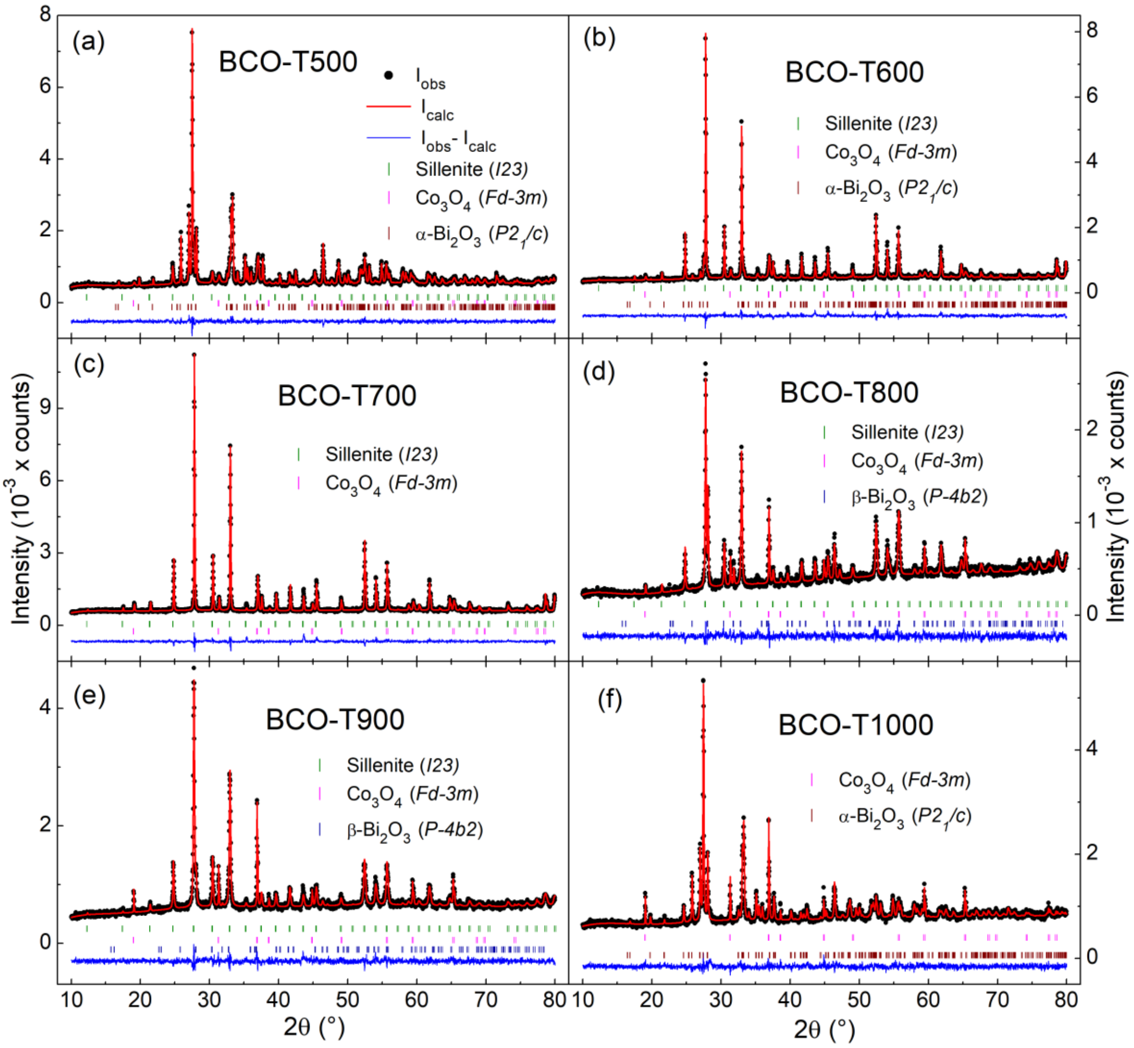

Fig. S1. X-ray diffractograms of BCO composites refined on GSAS: (a) BCO-T500, (b) BCO-T600, (c) BCO-T700, (d) BCO-T800, (e) BCO-T900 and (f) BCO-T1000.

S.1.3. Scanning electronic microscopy

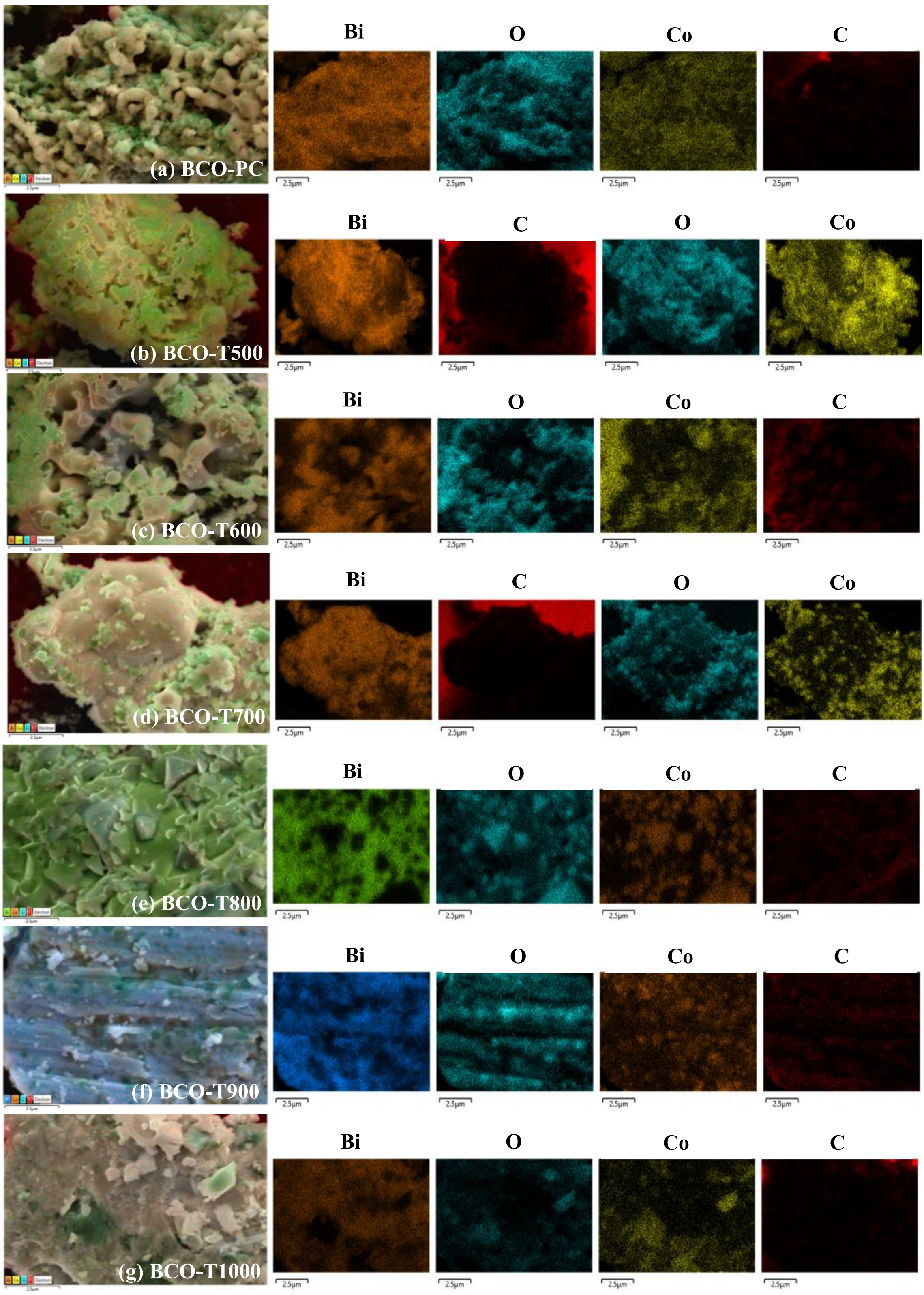

Fig. S2. SEM-EDS elemental map of BCO composites: (a) BCO-PC, (b) BCO-T500, (c) BCO-T600, (d) BCO-T700, (e) BCO-T800, (f) BCO-T900 and (g) BCO-T1000.

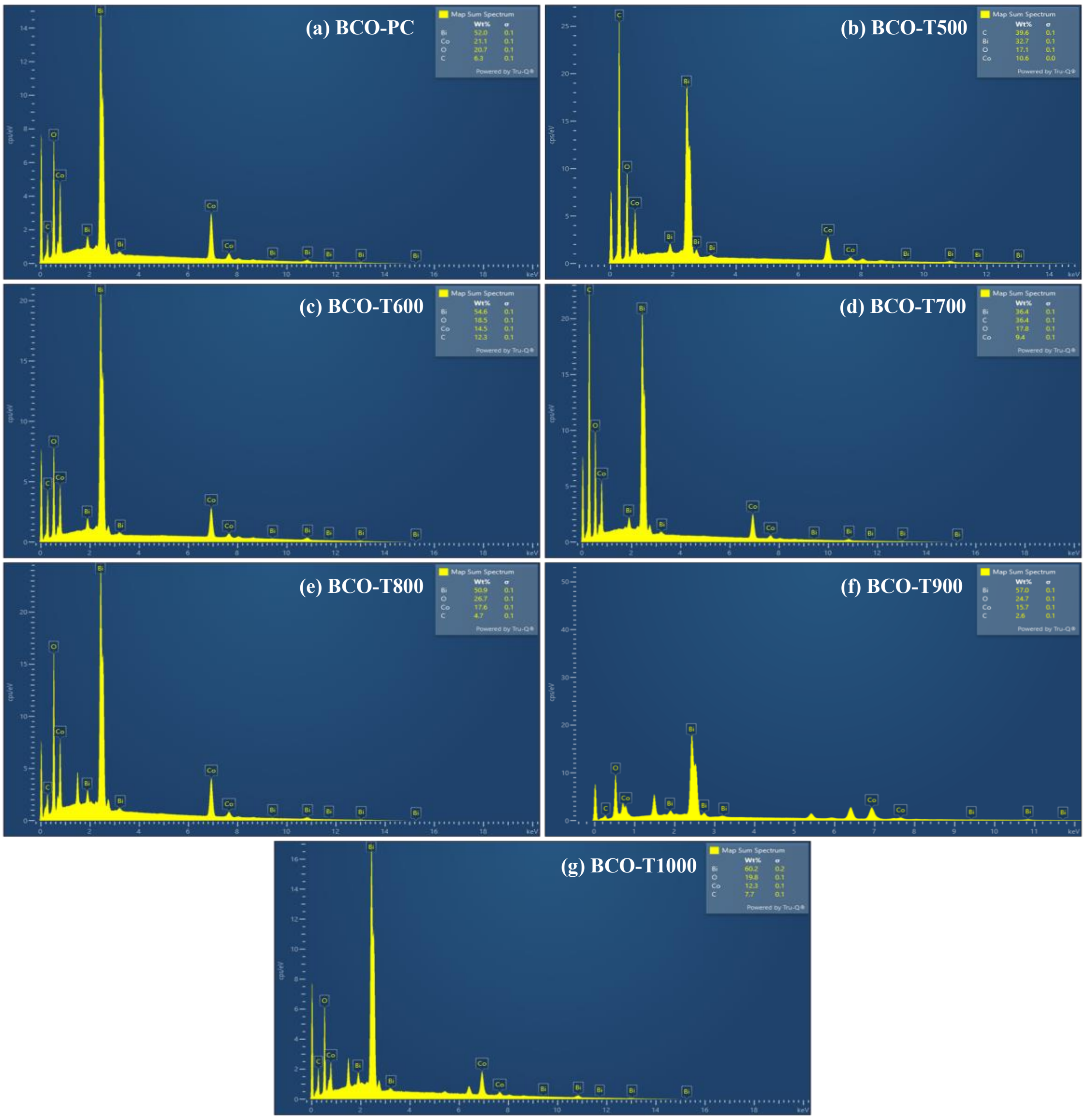

Fig. S3. SEM-EDS spectra of BCO composites: (a) BCO-PC, (b) BCO-T500, (c) BCO-T600, (d) BCO-T700, (e) BCO-T800, (f) BCO-T900 and (g) BCO-T1000.

Table S3. Atomic percentage (at%) of BCO materials obtained in SEM-EDS.

Sample	C	Bi	O	Co	Bi:Co:O
BCO-PC	21.64	10.26	53.33	14.77	1:1.4:5.2
BCO-T500	70.13	3.33	22.71	3.82	1:1.1:6.8
BCO-T600	38.12	9.72	43.00	9.16	1:0.9:4.3
BCO-T700	67.71	3.89	24.83	3.56	1:0.9:6.4
BCO-T800	15.05	9.36	64.11	11.48	1:1.2:6.8
BCO-T900	9.42	11.86	67.12	11.59	1:1:5.8
BCO-T1000	27.00	12.13	52.08	8.79	1:0.7:4.3

S.1.4. X-ray photoelectron spectroscopy

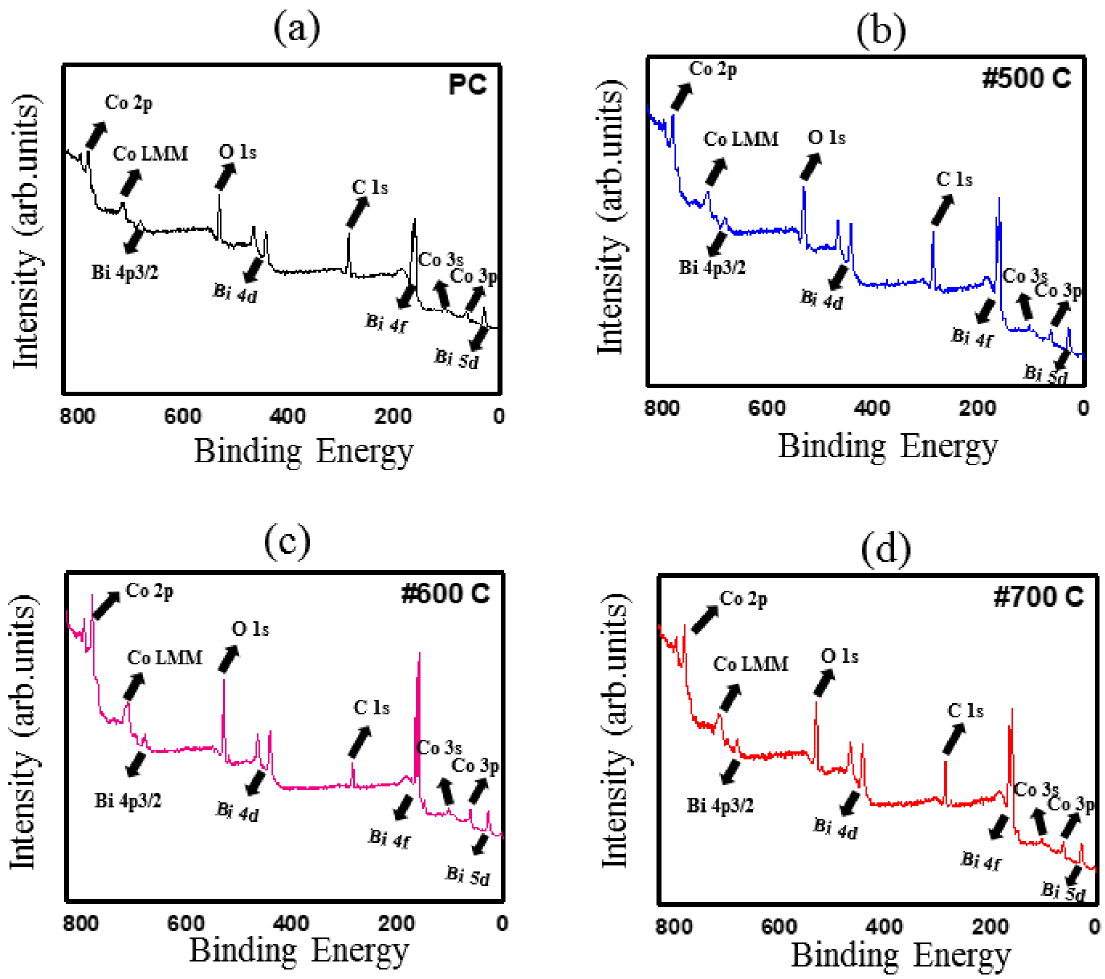

Fig. S4. XPS survey spectra of (a) BCO-PC, (b) BCO-T500, (c) BCO-T600 and (d) BCO-T700°C. All the remarkable features are pointed by black arrows.

S.1.5. Fourier transform infrared spectroscopy

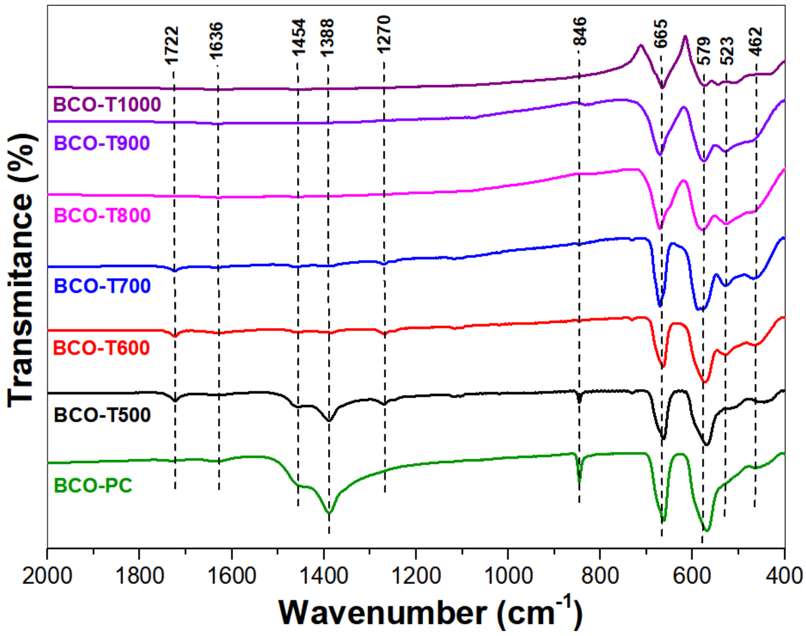

Fig. S5. FTIR spectra of sillenite composites.

Table S4. Assignment of FTIR band frequencies observed for BCO samples, along with their comparison to literature data.

$\bar{\nu}$ (cm^{-1})	Mode assignment	References
462	Bi–O vibration	1
523	Bi–O vibration	1
579	Bi–O–Co stretching	2,3
665	Co–O vibration	2,3
846	CO_3^{2-} vibration	4
1270	C-C(=O)-O stretching	5,6
1388	NO_3^- vibration	2,3
1454	NO_3^- vibration	2,3
1636	H–O–H stretching	7
1722	Assymmetric stretching of C=O	5

S.1.6. Raman spectroscopy

Table S5. Raman spectroscopy vibrational modes of BCO composites.

Raman shift (cm^{-1})	Assigned phase	Vibrational mode assignment	References
124	β-Bi_2O_3	—	8,9
148	Sillenite	Breathing of Bi and O atoms	3
197	Sillenite and Bi_2O_3	Bi–O	2,3
315	β-Bi_2O_3	—	8,9
453	β-Bi_2O_3	—	8,9
474	Sillenite	CoO_4 (tetrahedral site)	2,3
484	Co_3O_4	—	10
523	Sillenite	Bi–O Breathing of O atoms.	2,3
620	Sillenite	Vibrations and weak BiO	2,3
694	Co_3O_4	—	10

BCO-PC	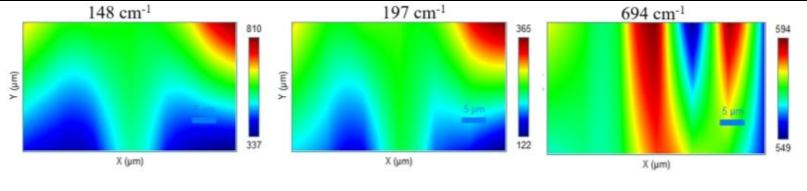
BCO-T500	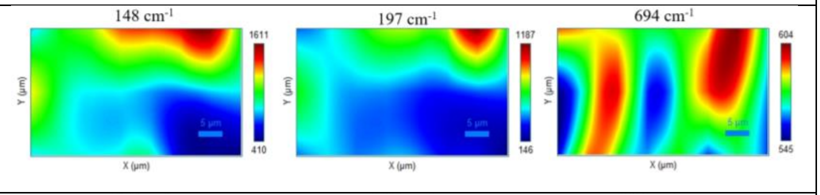
BCO-T600	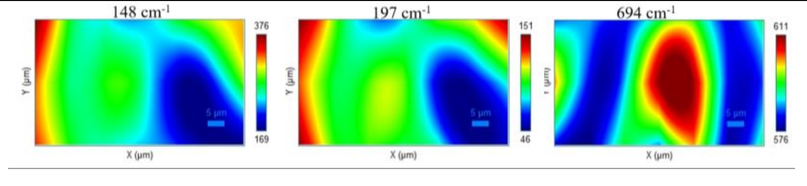
BCO-T700	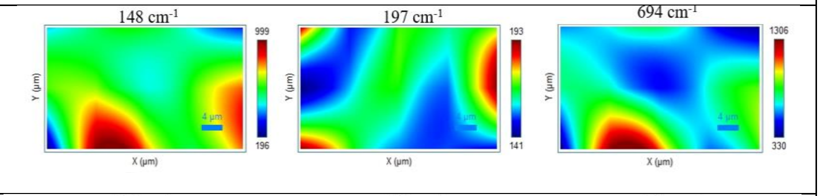
BCO-T800	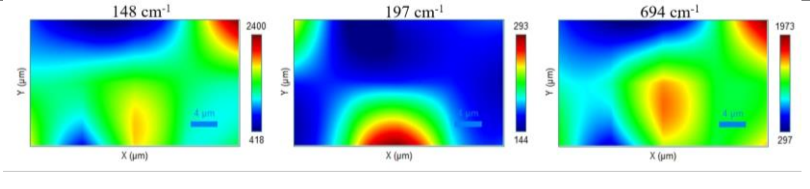
BCO-T900	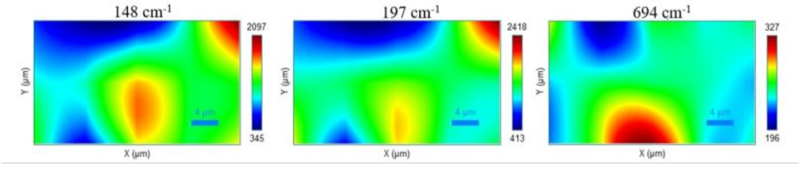
BCO-T1000	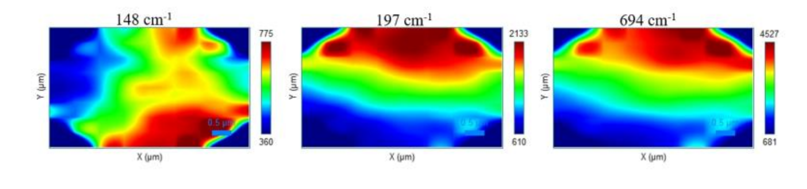

Fig. S6. Raman spectra mapping of BCO composites. The mapping bands were assigned as follows: and 148 cm^{-1} (sillenite/ Bi_2O_3), 197 cm^{-1} (sillenite/ Bi_2O_3) and 694 cm^{-1} (Co_3O_4).

S.2. Electrochemical analysis

S.2.1. Electrochemical impedance spectroscopy

Table S6. Effective capacitance obtained for the impedance spectra of sillenites.

Material	C_{eff}		
	R_{P1} (μF)	R_{P2} (μF)	R_{P3} (μF)
BCO-PC	4.56	18.5	208.0
BCO-T500	42.8	924.0	-
BCO-T600	8.22	130.0	26.8
BCO-T700	5.71	129.0	0.147

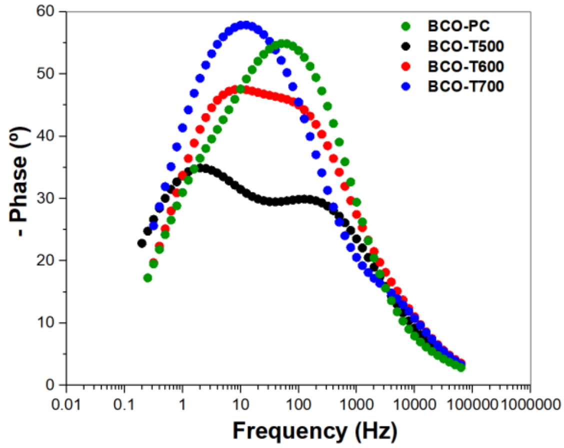

Fig. S7. Bode plot of the sillenite composites.

S.2.2. Linear sweep voltammetry

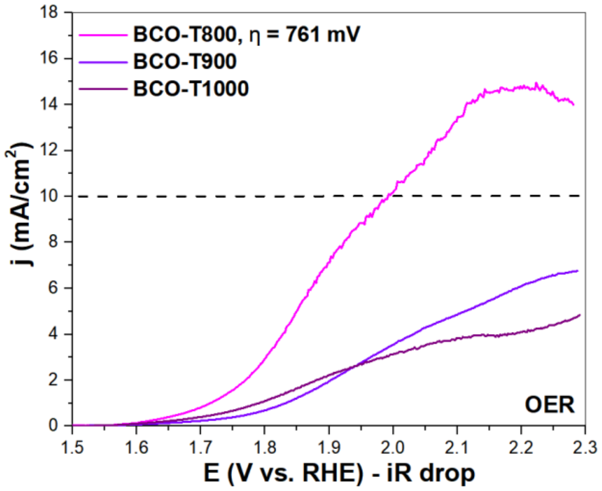

Fig. S8. Linear sweep voltammogram toward the OER with 85% iR compensation for the GC modified with BCO samples calcinated in the range of 800 – 1000 ºC.

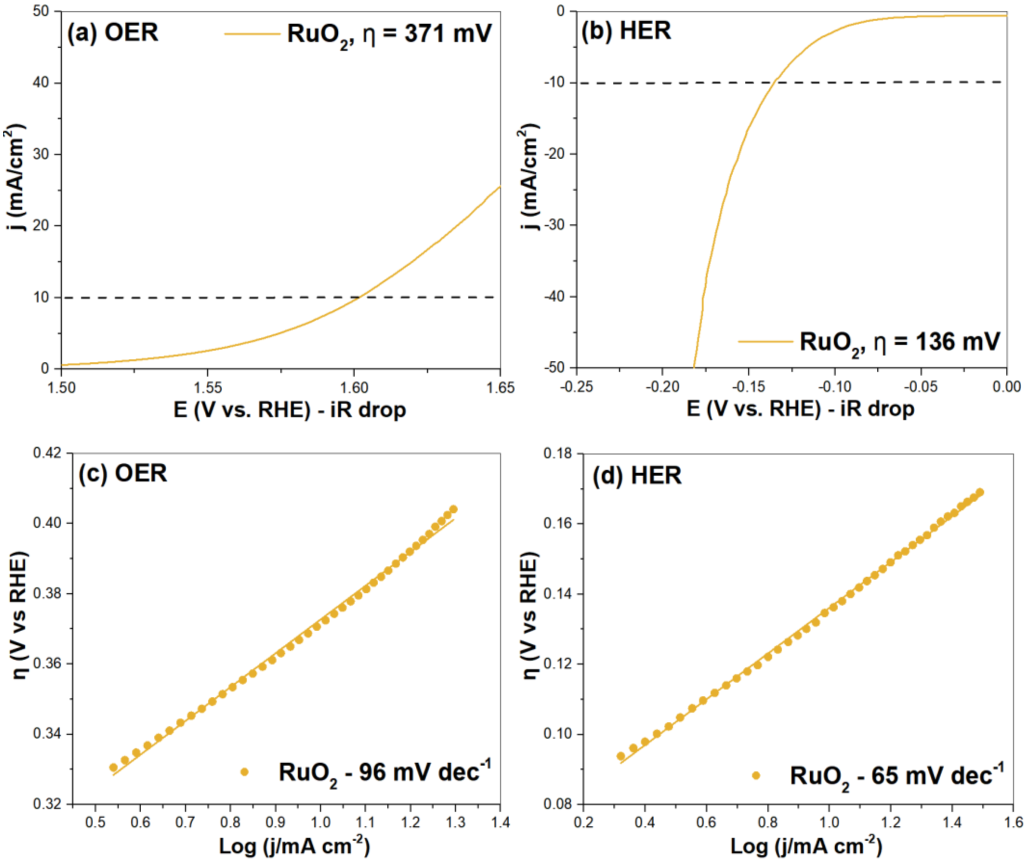

Fig. S9. Linear sweep voltammogram toward the (a) OER and (b) HER with 85% iR compensation for the GC modified with commercial RuO; Tafel plot of (c) OER and (d) HER.

S.2.3. Cyclic voltammetry

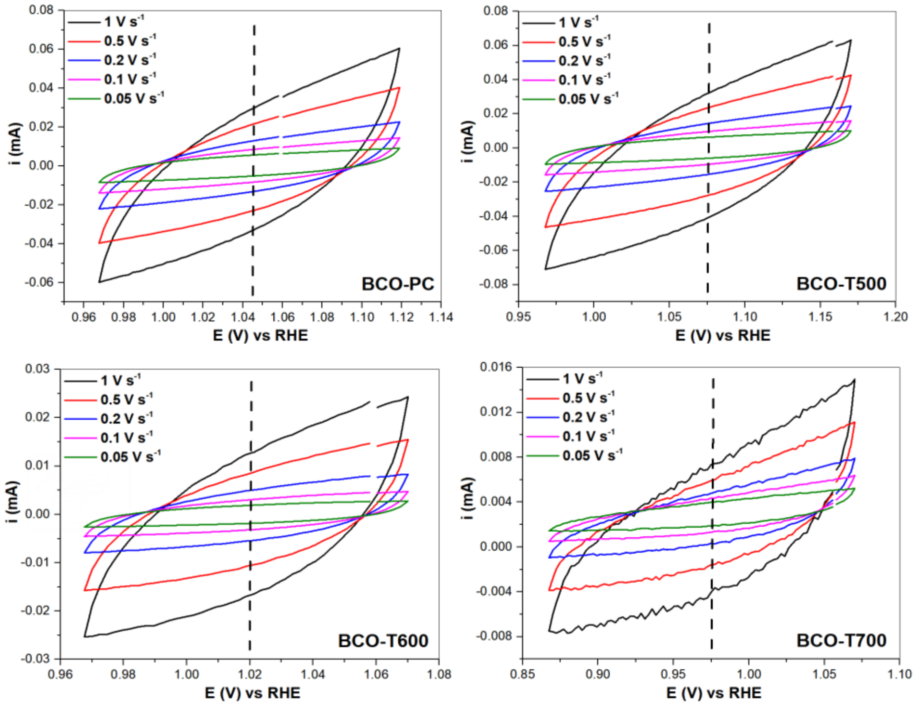

Fig. S10. CVs in the non-faradaic region of the samples at different scan rates from 0.05 to 1 V s^{-1}.

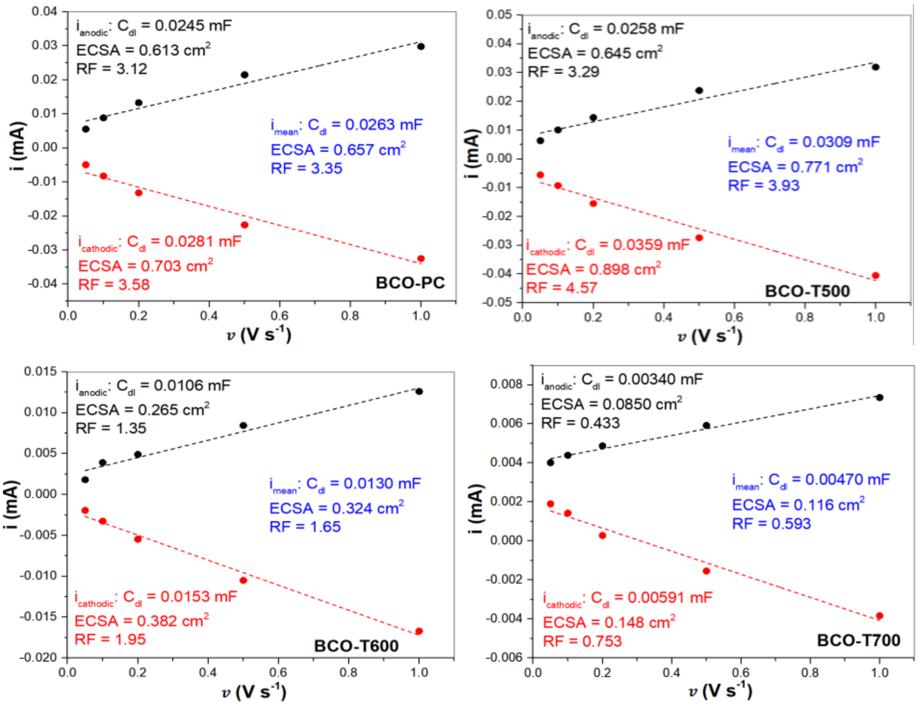

Fig. S11. Values of the electrochemical double layer capacitance (C_{dl}) and ECSA calculated from CVs data in **Fig. S10**.

S.2.4. Chronopotentiometric stability tests

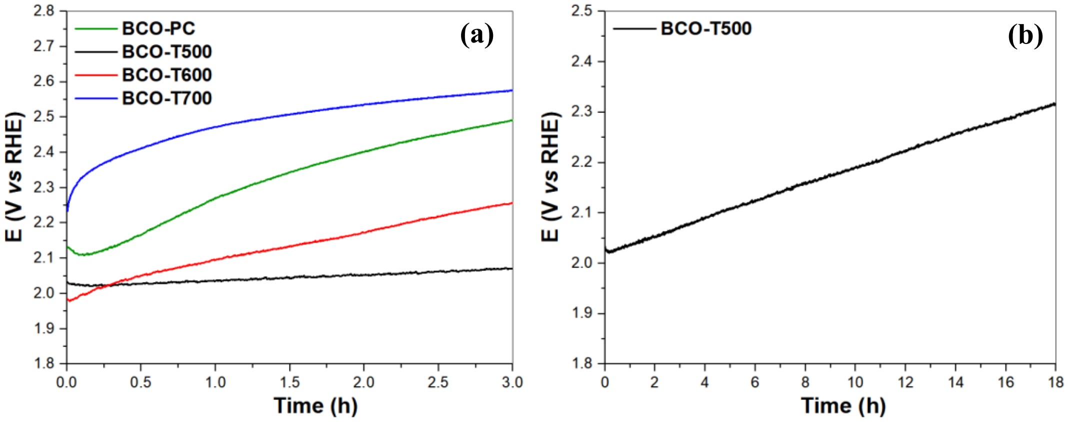

Fig. S12. (a) Chronopotentiometric stability tests for the OER of the FTO modified with BCO-PC, BCO-T500, BCO-T600 and BCO-T700 films performed at a constant current density of 10 mA cm^{-2} in 1 mol L^{-1} KOH (pH 14) over 3 h; (b) Long-term stability electrolysis of the BCO-T500 film performed at a constant current density of 10 mA cm^{-2} in 1 mol L^{-1} KOH (pH 14) over 18 h.

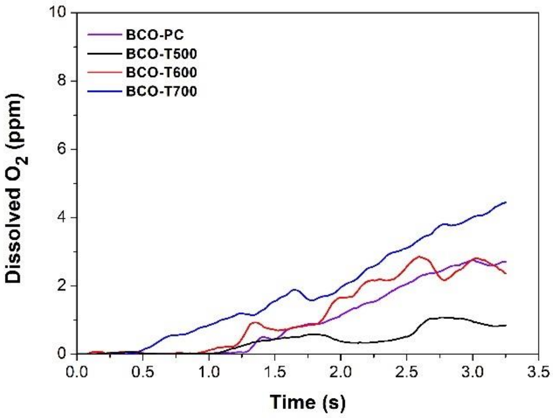

Fig. S13. Oxygen evolution determined by dissolved oxygen probe method during the chronopotentiometric stability tests of the FTO modified with BCO-PC, BCO-T500, BCO-T600 and BCO-T700 films performed at a constant current density of 10 mA cm^{-2} in 1 mol L^{-1} KOH (pH 14) over 3 h.

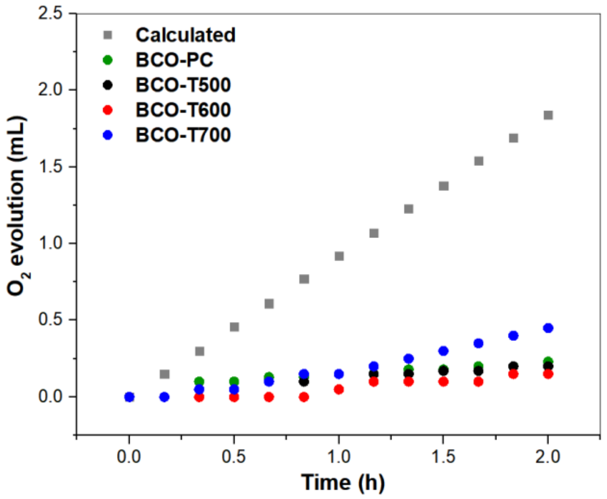

Fig. S14. Oxygen evolution determined by volumetry method during the chronopotentiometric stability tests of the FTO modified with BCO-PC, BCO-T500, BCO-T600 and BCO-T700 films, using an H-cell. with Faradaic efficiency measured vs predicted by Faraday equation performed at a constant current density of 10 mA cm^{-2} in 1 mol L^{-1} KOH (pH 14) for 2.5 h.

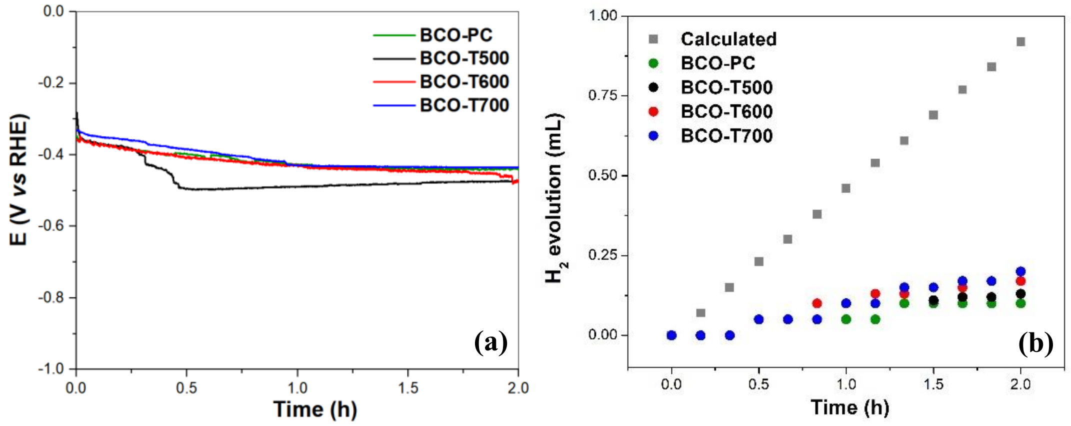

Fig. S15. (a) Chronopotentiometric stability tests for HER of the FTO modified with BCO-PC, BCO-T500, BCO-T600 and BCO-T700 films in FTO performed at a constant current density of -5 mA cm^{-2} in 1 mol L^{-1} KOH (pH 14) for 2 h, in a H-cell; (b) Hydrogen evolution monitored by volumetry during the stability tests, along with the corresponding Faradaic efficiencies compared to the theoretical values calculated from Faraday's law under the same operating conditions.

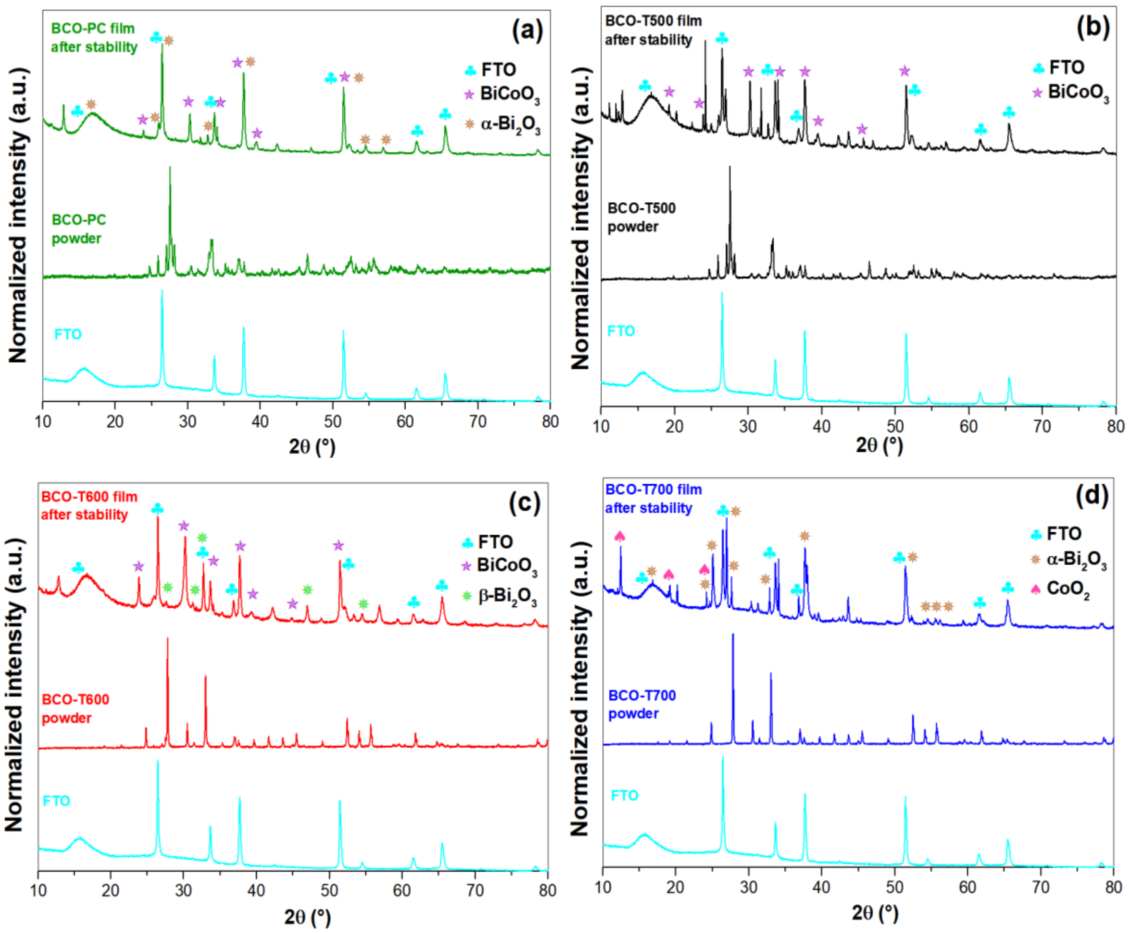

Fig. S16. Normalized X-ray diffractograms of BCO composites after stability tests of OER: (a) BCO-PC, (b) BCO-T500, (c) BCO-T600 and (d) BCO-T700. The peaks of the new phases formed after stability are marked in the diffractograms.

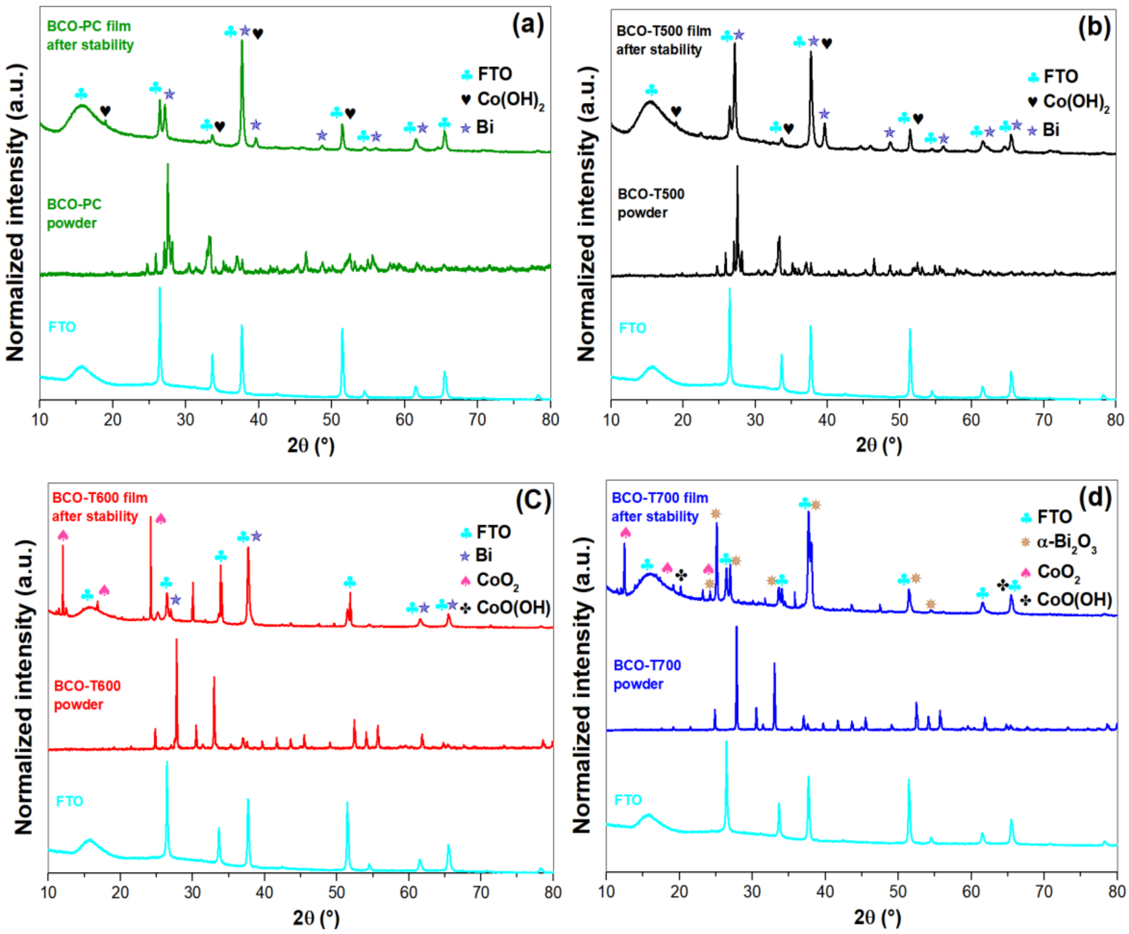

Fig. S17. Normalized X-ray diffractograms of BCO composites after stability tests of HER: (a) BCO-PC, (b) BCO-T500, (c) BCO-T600 and (d) BCO-T700. The peaks of the new phases formed after stability are marked in the diffractograms.

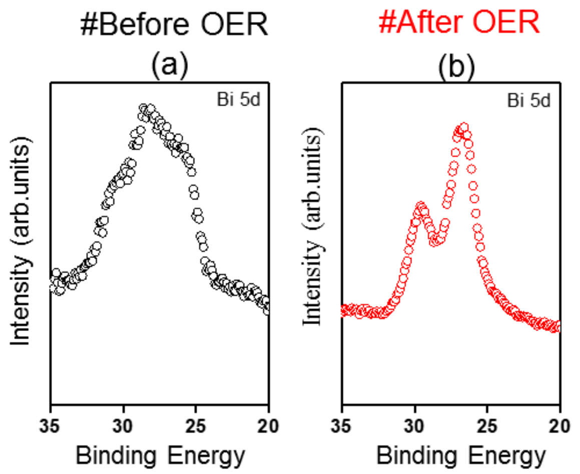

Fig. S18. Bi 5d spectra (a) before and (b) after OER experiments for the FTO modified BCO-T700 films.

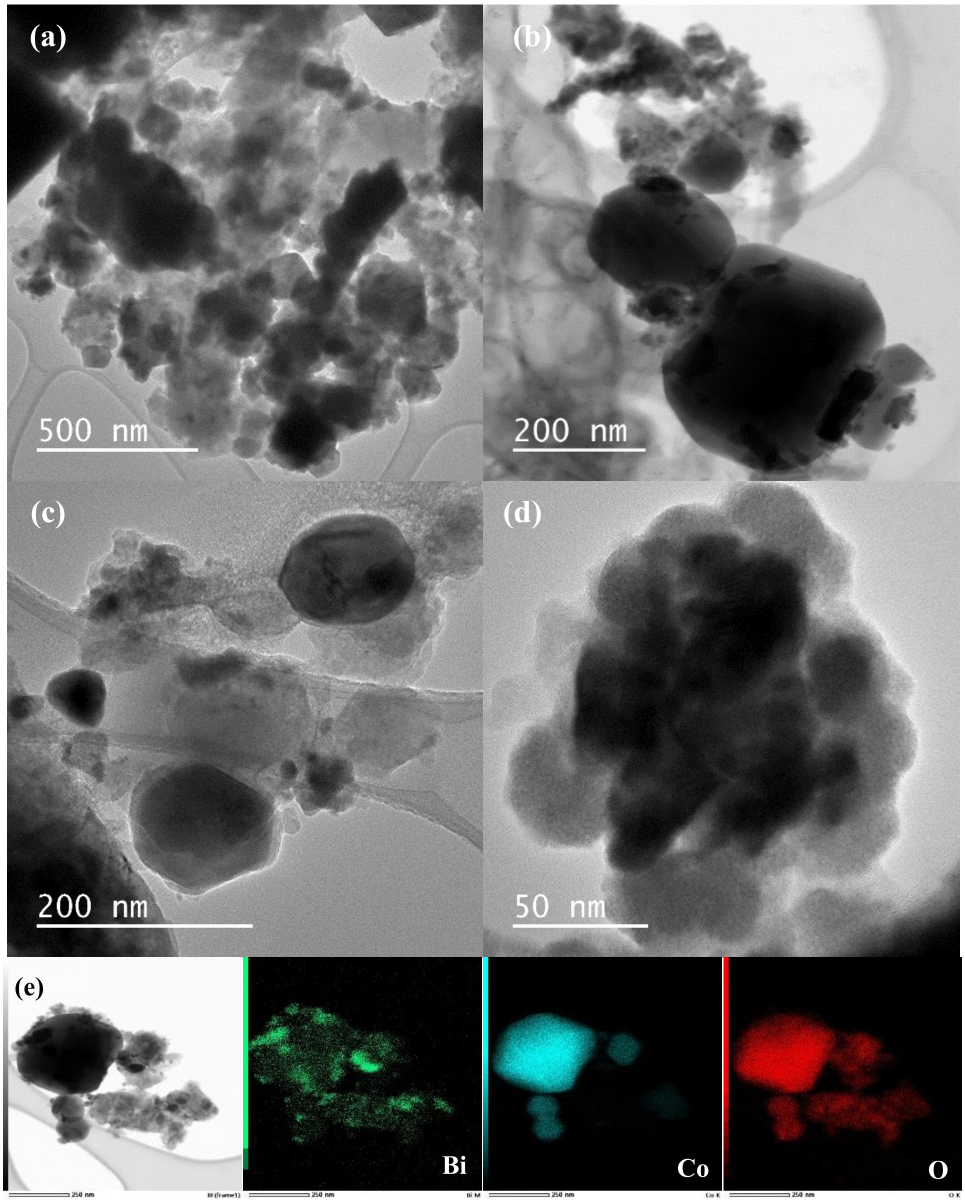

Fig. S19. (a, b, c, d) High magnification TEM micrograph of BCO-T700 after chronopotentiometric stability tests performed at a constant current density of 10 mA cm^{-2} in 1 mol L^{-1} KOH (pH 14) for 2 h; (e) TEM-EDS mapping of the BCO-T700 after the stability test of OER.

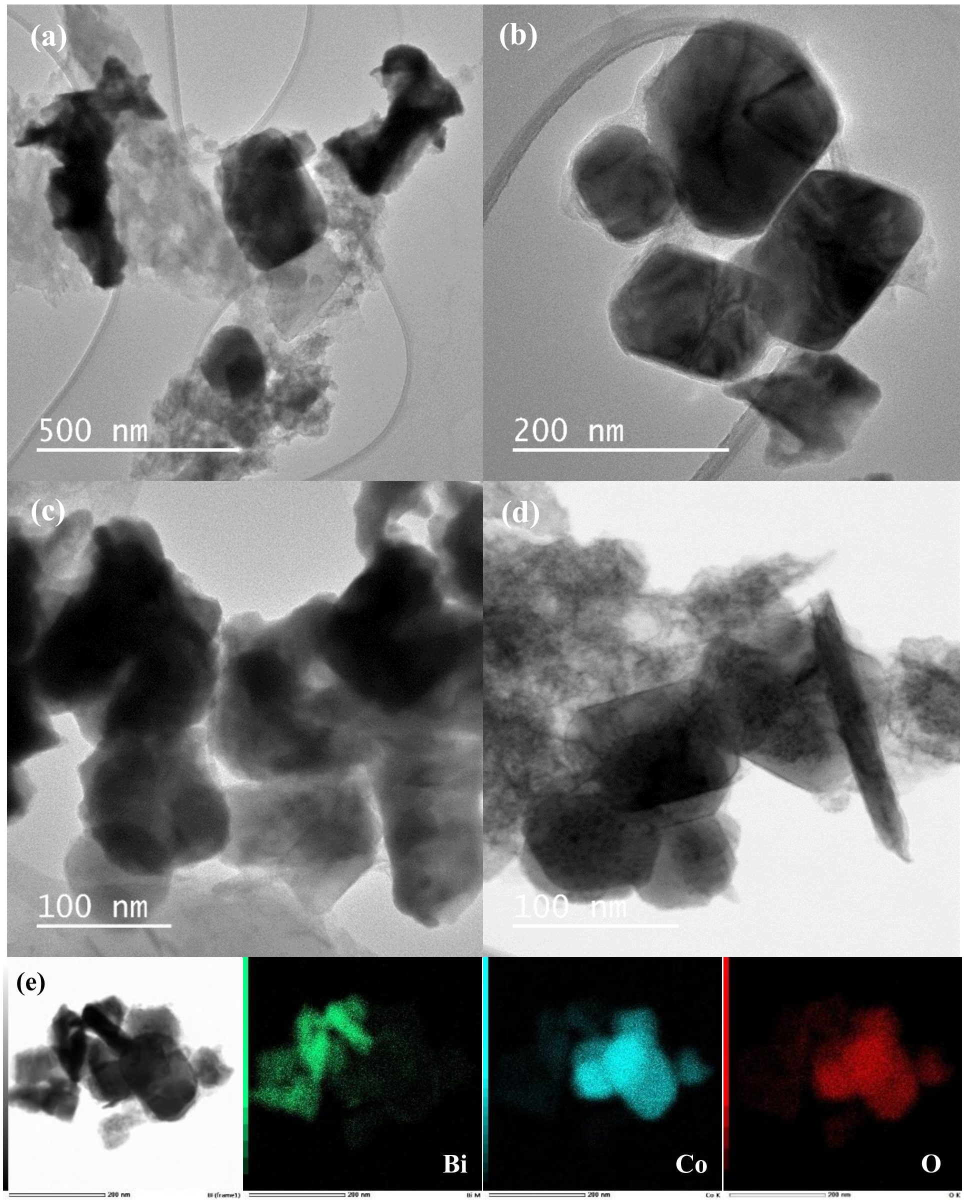

Fig. S20. (a, b, c, d) High magnification TEM micrograph of BCO-T700 after chronopotentiometric stability tests performed at a constant current density of -5 mA cm^{-2} in 1 mol L^{-1} KOH (pH 14) for 2 h; (e) TEM-EDS mapping of the BCO-T700 after the stability test of HER.

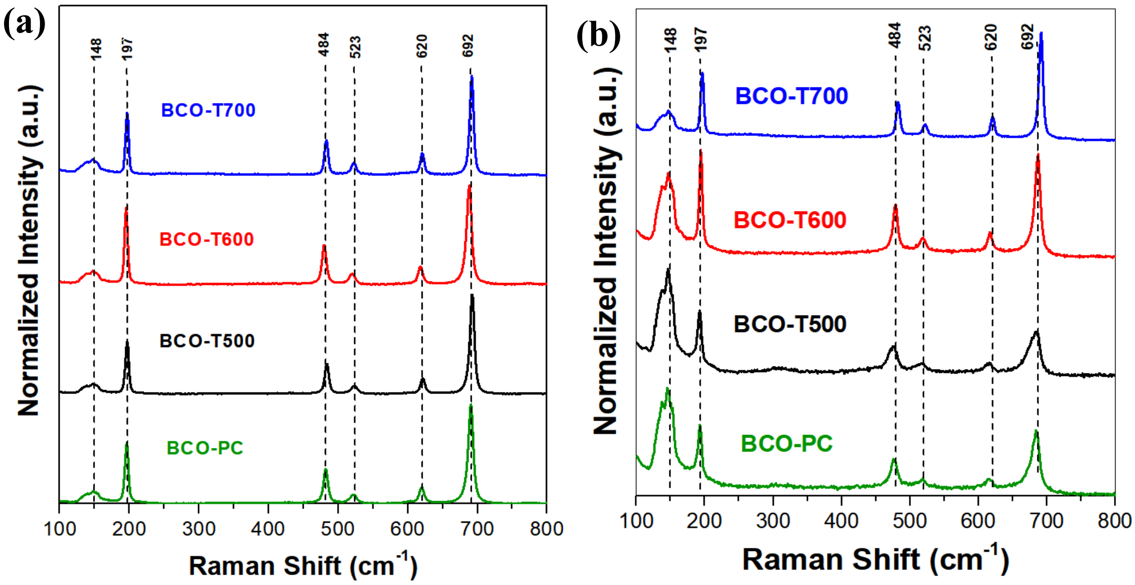

Fig. S21. Normalized Raman spectra of BCO composites after stability test performed by chronopotentiometry at: (a) 10 mA cm^{-2} for OER for 3 h and (b) -5 mA cm^{-2} for HER for 2 h in 1 mol L^{-1} KOH.

S.2.5. Overall water splitting tests

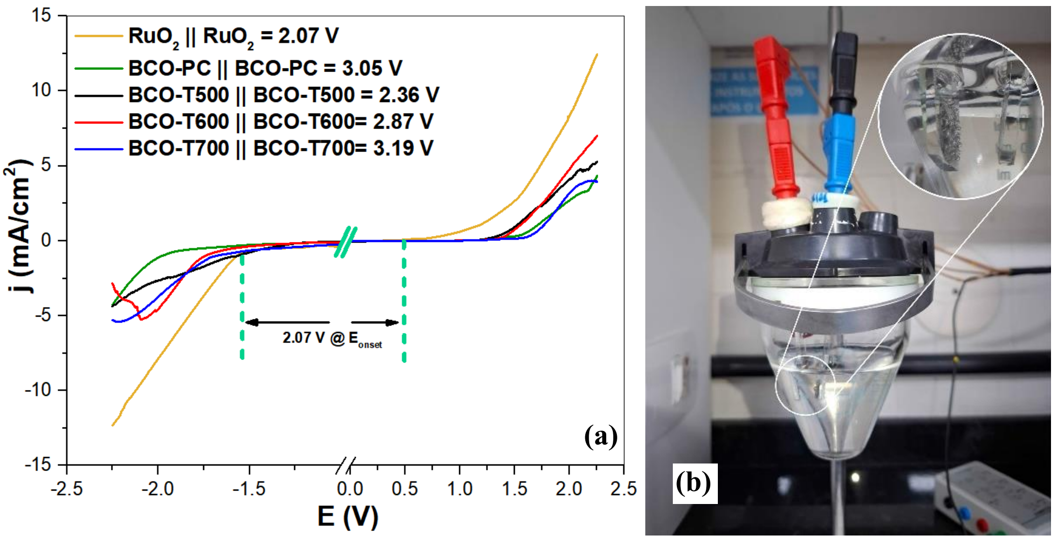

Fig. S22. (a) Electrochemical performances of the overall water splitting using the BCO electrocatalysts and commercial RuO_2 as both anode and cathode in a two-electrode configuration in in 1 mol L^{-1} KOH (pH 14) using FTO substrate. (b) Demonstration of electrochemical cell using two-electrode setup with hydrogen and oxygen gas evolution from the electrodes.

S.3. OER and HER performance of reported electrocatalysts

Table S7. Comparison of the performance of Bi and Co-based catalysts reported for OER and HER.

Material	Synthesis method	Electrolyte	η_{OER} (mV)/ j (mA cm^{-2})	η_{HER} (mV)/ j (mA cm^{-2})	References
$(Bi_{18}Co_6)Co_2O_{40}$—Co_3O_4 (BCO-T700)	Sol-gel	1.0 M KOH	475 mV/10 mA cm^{-2}	307 mV/−10 mA cm^{-2}	This work
$Bi_{25}FeO_{40}$	Hydrothermal	0.1 M KOH	640 mV/1 mA cm^{-2}	—	Vijay *et al.* (2022)[11]
$Bi_{24}Fe_2O_{39}$	Precipitation	1.0 M KOH	420 mV/10 mA cm^{-2}	—	Arora *et al.* (2024)[12]
$(Bi_{0.5}Co_{0.5})_2O_3$	Hydrothermal	0.1 M KOH	367 mV/10 mA cm^{-2}	—	LIU, H. *et al.* (2020)[13]
$BiCoO_3$	Solid state	0.1 M KOH	303 mV/10 mA cm^{-2}	—	Hu *et al.* (2023)[14]
$Bi_{0.07}Co_{2.93}O_4$	Precipitation	0.1 M KOH	350 mV/10 mA cm^{-2}	—	Gorylewski *et al.* (2025)[15]
Co_3O_4	Hydrothermal	1.0 M KOH	380 mV/10 mA cm^{-2}	—	ALEX *et al.* (2020)[16]
Co_3O_4 nanooctahedron with {111} crystal planes	Hydrothermal	1.0 M KOH	—	195 mV/−10 mA cm^{-2}	Liu Li *et al.* (2017)[17]
Co_3O_4	Hydrothermal	1.0 M KOH	301 mV/10 mA cm^{-2}	77.9 mV/−10 mA cm^{-2}	Wu *et al.* (2018)[18]
Bi_2O_3@BiNF*	Hydrothermal	1.0 M KOH	—	250 mV/−10 mA cm^{-2}	SYAH *et al.* (2021)[19]
Bi_2O_3@Ni foam	Hydrothermal	1.0 M KOH	—	174 mV/-10 mA cm^{-2}	Wu *et al.* (2022)[20]
Bi_2O_3@Ni Foam	Hydrothermal	1.0 M KOH	—	127 mV/-10 mA cm^{-2}	Wu *et al.* (2022)[21]

*Support material made from metallic Bi deposited on nickel foam.

S.4. References

(1) Muthu Kumar, A.; Ragavendran, V.; Mayandi, J.; Ramachandran, K.; Jayakumar, K. Enhancing Supercapacitance and Energy Density with Phase Tuning Agent: Bi25FeO40 Microcubes Perspective. Mater. Sci. Semicond. Process. **2024**, 177, No. 108357. DOI: 10.1016/j.mssp.2024.108357.

(2) Lopes Matias, J. A.; Silva, I. B. T.; da Silva, A. O.; Oliveira, J. B. L.; Ribeiro da Silva, D.; Morales, M. A. (Bi13Co11)Co2O40–Co3O4 Nanocomposites: Approach to Different Fuels in Sol-Gel Combustion Synthesis Using the Box-Behnken Design. Ceram. Int. **2022**, 48 (1), 481–494. DOI: 10.1016/j.ceramint.2021.09.124.

(3) Lopes Matias, J. A.; Sabino da Silva, E. B.; Raimundo, R. A.; Ribeiro da Silva, D.; Oliveira, J. B. L.; Morales, M. A. (Bi13Co11)Co2O40–Co3O4 Composites: Synthesis, Structural and Magnetic Properties. J. Alloys Compd. **2021**, 852, No. 156991. DOI: 10.1016/j.jallcom.2020.156991.

(4) Mechay, A.; Feki, H. E. L.; Schoenstein, F.; Jouini, N. Nanocrystalline Hydroxyapatite Ceramics Prepared by Hydrolysis in Polyol Medium. Chem. Phys. Lett. **2012**, 541, 75–80. DOI: 10.1016/j.cplett.2012.05.047.

(5) Gieroba, B.; Kalisz, G.; Krysa, M.; Khalavka, M.; Przekora, A. Application of Vibrational Spectroscopic Techniques in the Study of the Natural Polysaccharides and Their Cross-Linking Process. Int. J. Mol. Sci. 2023, 24 (3), No. 2630. DOI: 10.3390/ijms24032630.

(6) Matwijczuk, A.; Oniszczuk, T.; Matwijczuk, A.; Chruściel, E.; Kocira, A.; Niemczynowicz, A.; Wójtowicz, A.; Combrzyński, M.; Wiacek, D. Use of FTIR Spectroscopy and Chemometrics with Respect to Storage Conditions of Moldavian Dragonhead Oil. Sustainability 2019, 11 (22), No. 6414. DOI: 10.3390/su11226414.

(7) Liu, Y.; Guo, H.; Zhang, Y.; Tang, W.; Cheng, X.; Li, W. Heterogeneous Activation of Peroxymonosulfate by Sillenite Bi25FeO40: Singlet Oxygen Generation and Degradation for Aquatic Levofloxacin Chem. Eng. J. **2018**, 343, 128–137. DOI: 10.1016/j.cej.2018.02.125.

(8) Díaz-Guerra, C.; Almodóvar, P.; Camacho-López, M.; Camacho-López, S.; Piqueras, J. Formation of β-Bi2O3 and δ-Bi2O3 during Laser Irradiation of Bi Films Studied in-Situ by Spatially Resolved Raman Spectroscopy. J. Alloys Compd. **2017**, 723, 520–526. DOI: 10.1016/j.jallcom.2017.06.263.

(9) Depablos-Rivera, O.; Martínez, A.; Rodil, S. E. Interpretation of the Raman Spectra of Bismuth Oxide Thin Films Presenting Different Crystallographic Phases. J. Alloys Compd. **2021**, 853, No. 157245. DOI: 10.1016/j.jallcom.2020.157245.

(10) Li, Y.; Qiu, W.; Qin, F.; Fang, H.; Hadjiev, V. G.; Litvinov, D.; Bao, J. Identification of Cobalt Oxides with Raman Scattering and Fourier Transform Infrared

Spectroscopy. J. Phys. Chem. C **2016**, 120 (8), 4511–4516. DOI: 10.1021/acs.jpcc.5b11185.

(11) Jyoti, S.; Vijay, A.; Terranova, U.; Gupta, S. K.; Sudarshan, K.; Vaidya, S. Electrocatalytic OER Behavior of the Bi-Fe-O System: An Understanding from the Perspective of the Presence of Oxygen Vacancies. Phys. Chem. Chem. Phys. **2024**, 26 (24), 17324–17333. DOI: 10.1039/d4cp00348a.

(12) Arora, A.; Wadhwa, R.; Yadav, K. K.; Ankush; Jha, M. Enhanced Electrochemical Oxygen Generation from Sillenite Phase of Bismuth Iron Oxide (Bi24Fe2O39) Ultrafine Particles Stabilised at Room Temperature. J. Electroanal. Chem. **2024**, 958, No. 118154. DOI: 10.1016/j.jelechem.2024.118154.

(13) Liu, H.; Li, X.; Peng, C.; Zhu, L.; Zhang, Y.; Cheng, H.; Cui, J.; Wu, Q.; Zhang, Y.; Chen, Z.; Zou, W.; Gu, W.; Huang, H.; Wang, J.; Ye, B.; Fu, Z.; Lu, Y. Activating the Lattice Oxygen in (Bi0.5Co0.5)2O3 by Vacancy Modulation for Efficient Electrochemical Water Oxidation. J. Mater. Chem. A **2020**, 8 (26), 13150–13159. DOI: 10.1039/d0ta03411h.

(14) Hu, Y.; Li, L.; Zhao, J.; Huang, Y. C.; Kuo, C. yang; Zhou, J.; Fan, Y.; Lin, H. J.; Dong, C. L.; Pao, C. W.; Lee, J. F.; Chen, C. Te; Jin, C.; Hu, Z.; Wang, J. Q.; Zhang, L. Large Current Density for Oxygen Evolution from Pyramidally-Coordinated Co Oxide. Appl. Catal. B: Environ. **2023**, 333, No. 122785. DOI: 10.1016/j.apcatb.2023.122785.

(15) Gorylewski, D.; Zasada, F.; Słowik, G.; Lofek, M.; Grzybek, G.; Tyszczuk-Rotko, K.; Kotarba, A.; Stelmachowski, P. Modulation of the Electronic Properties of Co3O4 through Bi Octahedral Doping for Enhanced Activity in the Oxygen Evolution Reaction. ACS Catal. **2025**, 15 (6), 4746–4758. DOI: 10.1021/acscatal.4c07911.

(16) Alex, C.; Sarma, S. C.; Peter, S. C.; John, N. S. Competing Effect of Co3+ Reducibility and Oxygen-Deficient Defects Toward High Oxygen Evolution Activity in Co3O4 Systems in Alkaline Medium. ACS Appl. Energy Mater. **2020**, 3 (6), 5439–5447. DOI: 10.1021/acsaem.0c00297.

(17) Liu, L.; Jiang, Z.; Fang, L.; Xu, H.; Zhang, H.; Gu, X.; Wang, Y. Probing the Crystal Plane Effect of Co3O4 for Enhanced Electrocatalytic Performance toward Efficient Overall Water Splitting. ACS Appl. Mater. Interfaces **2017**, 9 (33), 27736–27744. DOI: 10.1021/acsami.7b07793.

(18) Wu, K.; Shen, D.; Meng, Q.; Wang, J. Octahedral Co3O4 Particles with High Electrochemical Surface Area as Electrocatalyst for Water Splitting. Electrochim. Acta **2018**, 288, 82–90. DOI: 10.1016/j.electacta.2018.08.067.

(19) Syah, R.; Ahmad, A.; Davarpanah, A.; Elveny, M.; Ramdan, D.; Albaqami, M. D.; Ouladsmane, M. Incorporation of Bi2O3 Residuals with Metallic Bi as High Performance Electrocatalyst toward Hydrogen Evolution Reaction. Catalysts **2021**, 11 (9), No. 10999. DOI: 10.3390/catal11091099.

(20) Wu, Z.; Liao, T.; Wang, S.; Mudiyanselage, J. A.; Micallef, A. S.; Li, W.; O'Mullane, A. P.; Yang, J.; Luo, W.; Ostrikov, K.; Gu, Y.; Sun, Z. Conversion of Catalytically Inert 2D Bismuth Oxide Nanosheets for Effective Electrochemical Hydrogen Evolution Reaction Catalysis via Oxygen Vacancy Concentration Modulation. Nano-micro Lett. **2022**, 14 (1), No. 90. DOI: 10.1007/s40820-022-00832-6.

(21) Wu, Z.; Mei, J.; Liu, Q.; Wang, S.; Li, W.; Xing, S.; Bai, J.; Yang, J.; Luo, W.; Guselnikova, O.; O'Mullane, A. P.; Gu, Y.; Yamauchi, Y.; Liao, T.; Sun, Z. Phase Engineering of Dual Active 2D Bi2O3-Based Nanocatalysts for Alkaline Hydrogen Evolution Reaction Electrocatalysis. J. Mater. Chem. A **2022**, 10 (2), 808–817. DOI: 10.1039/d1ta09019d.
